# Tunneling Mechanisms of Quinones in Photosynthetic Reaction Center–Light Harvesting 1 Supercomplexes

**DOI:** 10.1002/smsc.202400188

**Published:** 2024-09-15

**Authors:** Ruichao Mao, Jianping Guo, Lihua Bie, Lu‐Ning Liu, Jun Gao

**Affiliations:** ^1^ Hubei Key Laboratory of Agricultural Bioinformatics College of Informatics Huazhong Agricultural University Wuhan 430070 China; ^2^ College of Chemical and Biological Engineering Zhejiang University Hangzhou 310058 China; ^3^ Institute of Systems, Molecular and Integrative Biology University of Liverpool Liverpool L69 7ZB UK

**Keywords:** electron transport, enhanced sampling, molecular dynamics simulations, photosynthesis, quinone/quinol

## Abstract

In photosynthesis, light energy is absorbed and transferred to the reaction center, ultimately leading to the reduction of quinone molecules through the electron transfer chain. The oxidation and reduction of quinones generate an electrochemical potential difference used for adenosine triphosphate synthesis. The trafficking of quinone/quinol molecules between electron transport components has been a long‐standing question. Here, an atomic‐level investigation into the molecular mechanism of quinol dissociation in the photosynthetic reaction center–light‐harvesting complex 1 (RC–LH1) supercomplexes from *Rhodopseudomonas palustris*, using classical molecular dynamics (MD) simulations combined with self‐random acceleration MD‐MD simulations and umbrella sampling methods, is conducted. Results reveal a significant increase in the mobility of quinone molecules upon reduction within RC–LH1, which is accompanied by conformational modifications in the local protein environment. Quinol molecules have a tendency to escape from RC–LH1 in a tail‐first mode, exhibiting channel selectivity, with distinct preferred dissociation pathways in the closed and open LH1 rings. Furthermore, comparative analysis of free energy profiles indicates that alternations in the protein environment accelerate the dissociation of quinol molecules through the open LH1 ring. In particular, aromatic amino acids form *π*‐stacking interactions with the quinol headgroup, resembling the key components in a conveyor belt system. This study provides insights into the molecular mechanisms that govern quinone/quinol exchange in bacterial photosynthesis and lays the framework for tuning electron flow and energy conversion to improve metabolic performance.

## Graphical abstract

This study performs an atomic‐level analysis on the molecular mechanism of quinol dissociation from the photosynthetic reaction center‐light‐harvesting complex 1 (RC‐LH1) complex in *Rhodopseudomonas palustris*, uncovering the process, patterns, and channel selectivity. It highlights the significance of LH1 opening in facilitating quinol trafficking, providing important insights into quinone/quinol transport in bacterial photosynthesis.

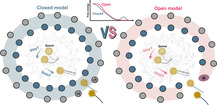

## 1 Introduction

**Figure 1:**
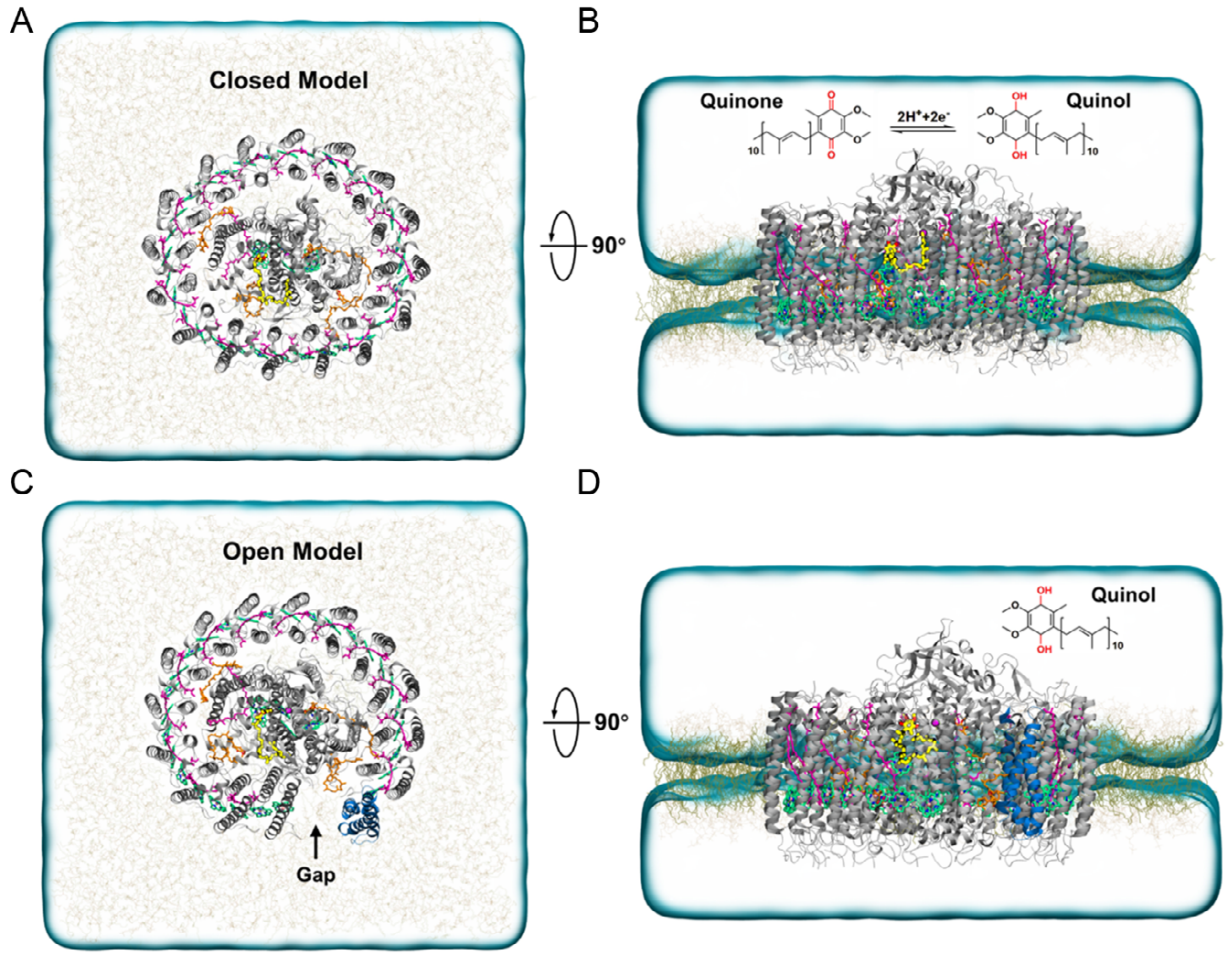
Schematic models of the simulations of RC–LH1. A,B) Schematic diagrams of the closed RC–LH1 model. C,D) Schematic diagrams of the open RC–LH1 model. All quinone molecules are shown in orange, except for the quinone molecule at the Q_B_ site, which is highlighted in yellow licorice. Protein chains are presented as gray cartoon models, with the protein‐W in the open ring model displayed in blue. The porphyrin rings of bacteriochlorophylls are shown in green, and carotenoids are depicted in purple.

In the photosynthetic process of purple bacteria, the solar energy absorbed by the light‐harvesting complex 1 (LH1) converges at the reaction center (RC), initiating the charge separation of specific bacteriochlorophyll pairs.^[^ ^1^ ^]^ Within an electron transfer chain composed of multiple cofactors, electrons are ultimately transferred to mobile carrier molecules known as quinones. Following the acquisition of two electrons and two protons from the cytoplasmic side, a quinone is reduced to a quinol (**Figure** **1**B). Subsequently, the quinol departs from the binding site of the RC and traverses the LH1 ring, transporting electrons and protons to a distant cytochrome *bc*_1_ complex. There, protons are released to the periplasmic side, establishing a proton gradient across the membrane to drive adenosine triphosphate synthesis. Simultaneously, electrons are transported back to the RC through cytochrome *c* ^[^ ^2^, ^3^ ^]^ or high‐potential iron–sulfur protein,^[^ ^4^, ^5^, ^6^ ^]^ thereby re‐engaging in the reduction of quinone.

The mobility of quinone/quinol molecules is crucial within the photosynthetic electron transfer chain, serving as a pivotal step that can limit the rate of energy conversion during photosynthesis. This mobility significantly influences the efficiency of converting light energy into usable forms. However, the molecular mechanism of quinone/quinol transport has been a longstanding question,^[^ ^1^, ^7^, ^8^ ^]^ due to the lack of high‐precision 3D structures and analytical approaches.

With the tremendous advancements of cryo‐electron microscopy (cryo‐EM) technology, many RC–LH1 supercomplex structures have been resolved to atomic‐level resolution, which facilitates the detailed analysis of the quinone/quinol exchange mechanism in detail.^[^ ^1^, ^7^, ^9^ ^]^ While the surrounding LH1 ring increases absorption cross section of the photosynthetic RC by accommodating pigments molecules and stabilizes the photosynthetic supercomplex, it also poses the question of how quinone molecules cross the LH1 barrier and transport electrons and protons from the RC to the cytochrome *bc*_1_ complex.^[^ ^10^ ^]^ The structures of RC–LH1 from *Thermochromatium (Tch.) tepidum,* ^[^ ^11^ ^]^ *Thiorhodovibrio (Trv.)* strain 970,^[^ ^12^ ^]^ and *Rhodospirillum* (*Rsp.*) *rubrum* ^[^ ^13^, ^14^ ^]^ revealed the closed LH1 rings assembled from 16 LH1 α/β subunits, presenting a greater challenge for the transfer progress of quinone molecules. Some purple bacteria have evolved specific mechanisms to facilitate quinone/quinol transport. For example, the LH1 complex of *Rhodobacter* (*Rba.*) *veldkampii* comprises 15 α/β subunits, forming an elliptical ring with a large opening.^[^ ^15^ ^]^ This opening, mediated by a transmembrane protein PufX, is suggested to facilitate quinone/quinol trafficking. The LH1 complex of *Blastochloris (Blc.) viridis* is composed of 16 sets of α/β/γ subunits and a set of α/β subunits arranged in an elliptical ring; the absence of the 17^th^ γ subunit creates a potential quinone diffusion channel.^[^ ^16^ ^]^ In *Rhodopseudomonas* (*Rps*.) *palustris*, both open and closed LH1 rings have been suggested to be present in native photosynthetic apparatus, depending on the presence or absence of a “protein‐W.”^[^ ^17^, ^18^ ^]^ The closed ring, as the dominant form, comprises 16 α/β subunits, whereas the open ring is formed by substituting two sets of α/β heterodimers in the closed ring with a protein‐W, accounting for ≈10% of total RC–LH1 complexes.^[^ ^18^ ^]^ The open and closed RC–LH1 complexes exhibited different levels of quinone dynamics.^[^ ^17^ ^]^ However, it remains unclear whether and how the closed and open LH1 ring structures play a role in mediating quinone/quinol diffusion.

Theoretical simulations based on the structural and spectroscopic information have demonstrated tremendous potential for investigating the mechanisms underlying the quinone/quinol exchange. Aird et al.^[^ ^19^ ^]^ examined the potential of mean force (PMF) along the diffusion pathway of quinone molecules through the hydrophobic pore generated by LH1 α/β subunits using steered molecule dynamics (SMD) simulations, which was further experimentally validated.^[^ ^20^ ^]^ In addition, microsecond‐scale all‐atom molecule dynamics (MD) simulations were performed based on the RC–LH1 structure of *Rba.* *veldkampii*, and three potential channels for quinone/quinol diffusion were inferred based on the local motion of quinone molecules.^[^ ^15^ ^]^ The first 100‐million atom‐scale model and MD simulations of an entire photosynthetic organelle from purple bacteria revealed a two‐state model for quinone molecules, denoted “swimming” and “diving,” respectively.^[^ ^21^ ^]^ Despite these efforts, the specific pathway, as well as thermodynamic and kinetic information regarding quinol dissociation in any RC–LH1 complex, is yet to be extensively elucidated.

In this study, we performed an atomic‐level analysis of the molecular mechanism of quinol dissociation within *Rps. palustris* RC–LH1 complexes, by applying classical MD simulations in conjunction with self‐random acceleration molecular dynamics‐molecular dynamics (S‐RaMD‐MD) simulations and umbrella sampling. Our results unveiled distinctions in the mobility of quinone molecules pre and postreduction, shedding light on the mode and channel selectivity of quinol dissociation in RC–LH1, as well as the significance of the LH1 opening in quinol dissociation. These findings provide insights into the mechanisms driving quinone/quinol transport in bacterial photosynthesis.

## 2 Experimental Section

### 2.1 Model Construction and Molecular Dynamics Simulation

The cryo‐EM structures of *Rps. palustris* RC–LH1 were selected as the initial structures for establishing the closed (PDB ID: 6Z5R) and open (PDB ID: 6Z5S) RC–LH1 systems.^[^ ^17^ ^]^ In the initial step, missing amino acid residues were added to both structures, and the missing secondary quinone (Q_B_) molecule from the open model was supplemented. Detailed information was provided in Supporting Information. Subsequently, the quinone molecule was modified to quinone (unprotonated, Q_B_) and quinol (double protonated, Q_B_H_2_) to simulate its oxidation and reduction states, respectively (Figure 1).

The phospholipid components of *Rps. palustris* chromatophores were primarily phosphatidylglycerol (PG: −1 charge), phosphatidylethanolamine (PE: neutral charge), cardiolipin (CL: −2 charge), phosphatidylcholine (PC: neutral charge), and hopanoids (HOP: neutral charge).^[^ ^22^ ^]^ However, the proportions of these components remained unknown. Given the dominant role of PC in the *Rps. palustris* RC–LH1 structures,^[^ ^17^ ^]^ we utilized 1‐palmitoyl‐2‐oleoyl‐sn‐glycero‐3‐phosphocholine (POPC) to mimic the local membrane environment of *Rps. Palustris* RC–LH1 in this study. The selection of a single type of lipids was aimed at simplifying the computational model and avoiding unnecessary complexity. Similar strategies were widely used in previous computational simulations of RC–LH1 complexes.^[^ ^23^, ^24^, ^25^ ^]^ First, the entire RC–LH1 complex was embedded into a pre‐equilibrated lipid bilayer composed of single‐component POPC, guided by the predicted phospholipid position of the Positioning of Proteins in Membrane server.^[^ ^26^ ^]^ To mimic the negatively charged environment as closely as possible to the native state, we retained all original lipid molecules identified in the cryo‐EM structure, including negatively charged phospholipids such as PG and CL. Therefore, while we only used POPC to fill small gaps, our system incorporated a diverse range of negatively charged phospholipids that fulfilled their respective functions. Subsequently, the system was solvated in a TIP3P water box, with dimensions of 200.9 × 182.0 × 115.3 Å^3^ for the closed model and 186.5 × 171.6 × 112.3 Å^3^ for the open model, respectively. Finally, to achieve neutralization, counterions (Cl^−^) was added, and 0.15 mm NaCl was introduced to simulate the physiological salt concentration.^[^ ^17^, ^27^ ^]^ The closed model contained a total of 249 Cl^−^ and 229 Na^+^, whereas the open model included a total of 212 Cl^−^ and 191 Na^+^. Figure 1 illustrates the resulting closed and open models of RC–LH1 within their respective physiological environments, containing 376 866 and 320 617 atoms, respectively.

The energy minimization of the entire system was performed in three successive steps. 1) The positions of all phospholipids, water molecules, and added residues were minimized with 20 000 steps using the steepest descent algorithm, while the remainder of the system was fixed; 2) A subsequent 20 000‐step energy minimization was performed for the whole system with restraints (100 kcal mol^−1^ Å^−2^) on the protein backbone (excluding the added residues) and heavy atoms of cofactors; 3) An additional 20 000‐step energy minimization was executed without any restraints. Subsequently, the whole system was gradually heated to 300 K for 120 ps. During this process, a restraint (10 kcal mol^−1^ Å^−2^) was applied to the protein backbone atoms, as well as the heavy atoms of the cofactors and phospholipid headgroups. Following these optimization and heating procedures, a 4 ns equilibrium simulation was performed under the constant number, pressure, and temperature NP ensemble at 300 K to stabilize the dimensions and density of the system, with all constraints gradually released during this process. Finally, a 50 ns unconstrained simulation was performed, where the atomic coordinates of all atoms were recorded every 1 ps. The MD simulations of the closed (quinone/quinol) model and the open (quinol) model were run three times independently, for a total of 450 ns.

All simulations were performed using NAMD 2.14.^[^ ^28^ ^]^ Temperature control utilized the Langevin thermostat,^[^ ^29^ ^]^ and the pressure was regulated using the anisotropic Langevin piston Nosé‐Hoover method.^[^ ^30^ ^]^ Bond lengths involving hydrogen atoms were constrained using the SHAKE algorithm.^[^ ^31^ ^]^ Van der Waals interactions were treated with a nonbonding cutoff of 10 Å, and electrostatic interactions were computed using the particle mesh Ewald method.^[^ ^32^ ^]^ Detailed information on the force field parameter settings for the molecules in the system is provided in Supporting Information.

### 2.2 S‐RaMD‐MD Simulation Scheme

**Figure 2:**
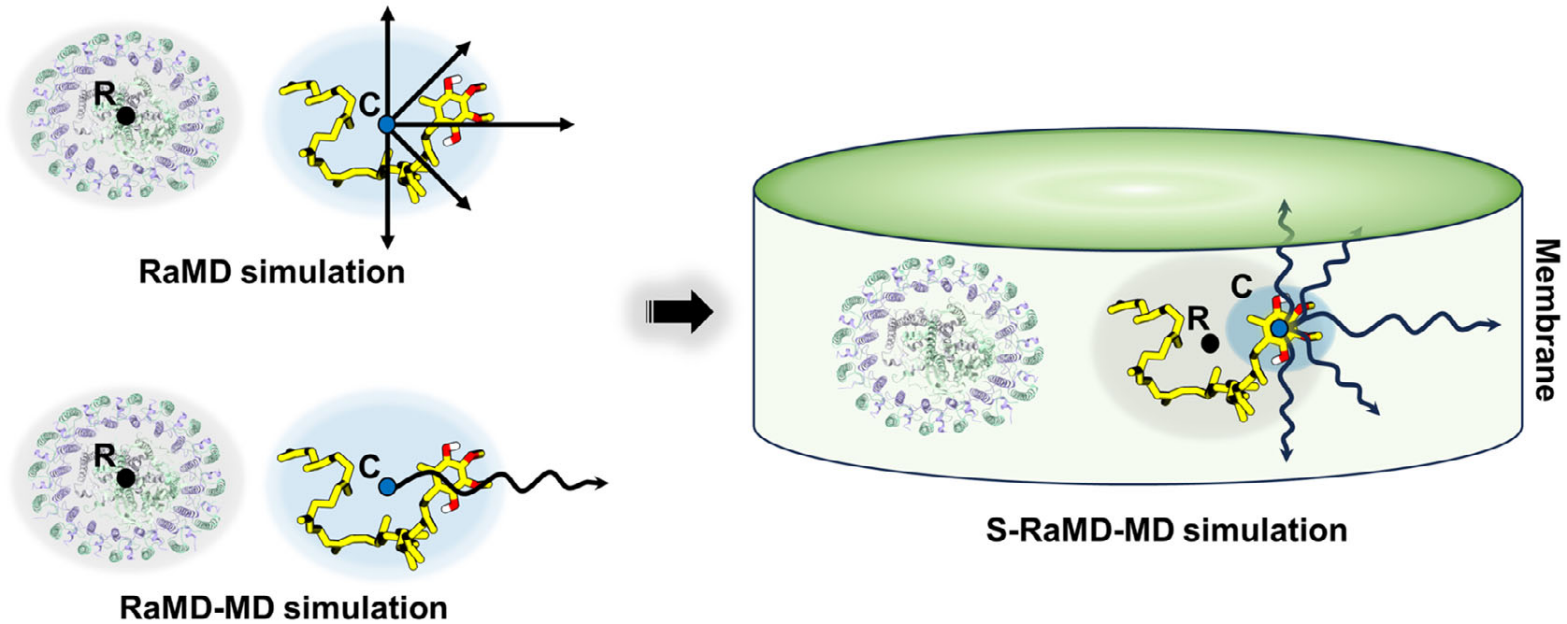
Principle of S‐RaMD‐MD simulation. The blue ellipse signifies the force application area, labeled as C; the gray ellipse represents the reference position, denoted as R. The straight arrow symbolizes a “reckless” forward movement, while the curved arrow signifies a “roundabout” forward movement. The green cylinder illustrates permissible range of movement for quinol molecules.

The inherent limitations pose challenges in providing a comprehensive description of the quinol dissociation process using the conventional RaMD or RaMD‐MD method (see Supporting Information for details). To address this, we introduced an S‐RaMD‐MD simulations scheme (**Figure** **2**), which capitalizes on the specific characteristics of quinol dissociation and integrates the strengths of RaMD and RaMD‐MD simulation algorithms. This offers improvements over the conventional method in several key aspects. First, we redefined the force‐applying atom (labeled C) as the head (or tail) of the quinone molecule, rather than the center of mass of the entire molecule. This adjustment facilitates the dissociation of the quinol molecule in a head‐first or tail‐first mode. Second, the reference site (labeled R) was defined as the center of mass of the quinol molecule itself, rather than the protein molecule. Simultaneously, the distance threshold D was set to a value greater than the length of the quinol molecule. This strategy encourages the head (or tail) of the quinol molecule to consistently move away from its center of mass, subsequently promoting random movement and dissociation. Additionally, to prevent the force‐exerting atoms from accidentally escaping the membrane environment, constraining forces are applied at the boundaries on both sides of the membrane. This allows force‐exerting atoms to move freely within the membrane, with constraining forces becoming active upon crossing the membrane boundary.

The initial structures for S‐RaMD‐MD simulations were derived from the equilibrium state trajectories of classical MD simulations, corresponding to snapshots at 40, 45, and 50 ns, respectively. For each initial structure, the acceleration (a) is selected as 0.3, 0.35, 0.4, 0.45, and 0.5 kcal Å^−1 ^g^−1^, while the distance threshold (*d*) is selected as 0.3, 0.4, and 0.5 Å, resulting in a total of 15 parameter combinations. The force‐exerting atoms (or accelerated atoms) for head‐first and tail‐first were set to the six‐carbon ring of the quinol head group and the last six carbon atoms in the tail group, respectively. The occurrence of a dissociation event was determined by tracking the passage of the center of mass of isoprene (C27‐C31) in the middle of the quinol molecule through the LH1 protein ring within a specific time frame (10 ns in this study). For the closed and open models, a total of 135 S‐RaMD‐MD simulations were conducted (Table S2 and S3, Supporting Information), totaling 1350 ns.

### 2.3 Umbrella Sampling

The umbrella sampling method was employed to quantify the free energy change during quinol dissociation and identify the most preferred channel. With a focus on the channel exhibiting the highest dissociation probability, the initial model for each window was constructed using a stable snapshot structure from the conventional MD simulation segment of the S‐RaMD‐MD simulation. The reaction coordinate was defined as the projection distance in the *xy*‐plane between the six‐carbon ring of the quinol head group and the Cα atom of a specific protein residue. Subsequently, for each window, 6 ns MD simulations were conducted with an appropriate biasing harmonic potential along the reaction coordinate to ensure reasonable overlap among all windows. For the three dissociation forms (head‐ or tail‐first dissociation in the closed model, tail‐first dissociation in the open model), the total simulation time for umbrella sampling reached 1,368 ns. Finally, the weighted histogram analysis method (WHAM) was implemented to calculate the probability distributions after equilibration and to generate the corresponding free energy profiles.

## 3 Results and Discussion

### 3.1 Conformational Changes in Quinones Following Reduction

**Figure 3:**
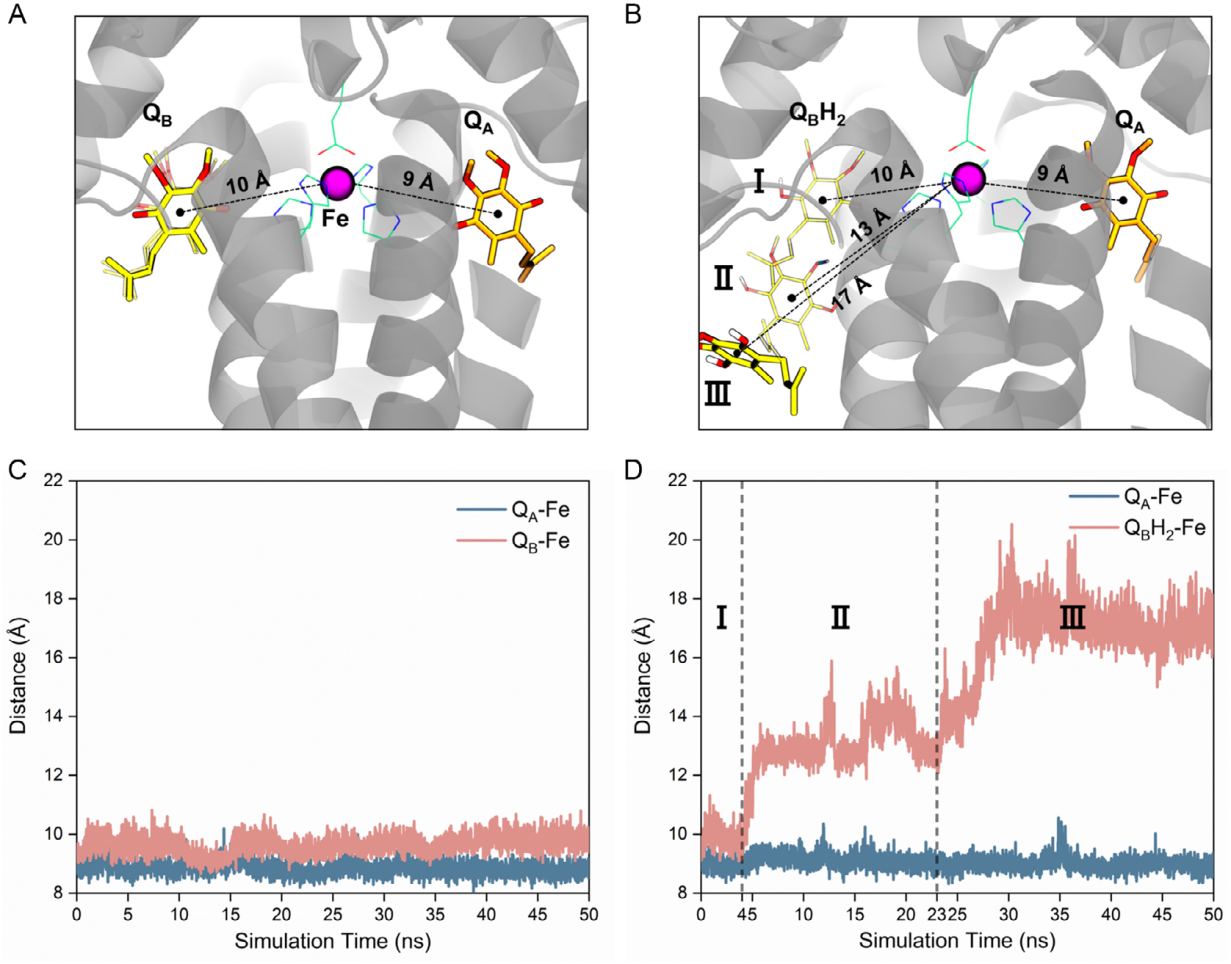
Differences in the mobility of quinones before and after reduction. A,B) The positional changes of quinone molecules before (Q_A_/Q_B_) and after (Q_A_/Q_B_H_2_) reduction, respectively. The protein is represented by gray cartoon model, Q_A_ is shown in orange, Q_B_ in yellow, the iron ion in purple, and its coordinating residues in green. C) and D) The distance changes between the six‐carbon ring of the quinone head groups and the nonheme iron before (Q_A_/Q_B_) and after (Q_A_/Q_B_H_2_) reduction, respectively.

A comparison of the MD simulation trajectories between the Q_B_ and Q_B_H_2_ models revealed a significant conformational change in quinone molecules after reduction (**Figure** **3**). To quantitatively assess conformational changes, the distances between the nonheme iron atoms and the center of mass of the six‐carbon ring of the quinone headgroups (Q_A_ and Q_B_ in the Q_B_ model; Q_A_ and Q_B_H_2_ in the Q_B_H_2_ model) were measured. The Q_A_ headgroups in both models remained stable at a distance of about 9 Å from the nonheme iron atom during the entire MD simulations, suggesting an enhanced level of structural stability compared to those of Q_B_ or Q_B_H_2_. This aligns with the function of Q_A_, which is responsible for single‐electron transfer without protonation during the photosynthetic reaction. The restricted displacement may be more conducive to the optimization of its function.

For quinone molecules at the Q_B_‐binding site, the distance was stably maintained at around 10 Å before reduction (Figure 3A,C) and changed to over 17 Å after reduction (Figure 3B,D). These results indicate that the headgroup of the reduced quinone underwent a conformational change and exited from the Q_B_ binding site. Based on the distance variation, the escape process of the quinol headgroup can be divided into three stages (Figure 3B,D). First, the distance fluctuated around 10 Å, and the escape process had not yet started. Second, the Q_B_H_2_ headgroup underwent a displacement of ≈3 Å within 2 ns (from 4 to 6 ns), after which the distance oscillated violently between 12 and 16 Å. Finally, the distance increased from 13 to 20 Å within 7 ns (from 23 to 30 ns) and then oscillated around 17 Å, maintaining this pattern until the end of the simulation. Such distance variations were consistently captured in three independent MD simulations (Figure S1, Supporting Information), distinctly indicating that the quinone molecule tends to stably bind at the Q_B_‐binding site, whereas the quinol molecule exhibited pronounced movement characteristics, tending to depart from the Q_B_‐binding site.

It is noteworthy that the protein environment surrounding the Q_B_ site also underwent conformational changes. After Q_B_ reduction, the mobility of the short loop situated between Helix de and Helix E (denoted as Loop_de‐E_) and near the Q_B_ binding site noticeably increased (Figure S2A, Supporting Information Results and Discussions). Meanwhile, Loop_de‐E_ exhibited a tendency to move away from the Q_B_H_2_ headgroup (Figure S2B, Supporting Information Results and Discussions). This suggests that, during the release of the Q_B_H_2_ headgroup, the conformation of Loop_de‐E_ underwent adaptive modifications, thereby facilitating the conformational changes of the Q_B_H_2_ molecule and creating space for its release.

**Figure 4:**
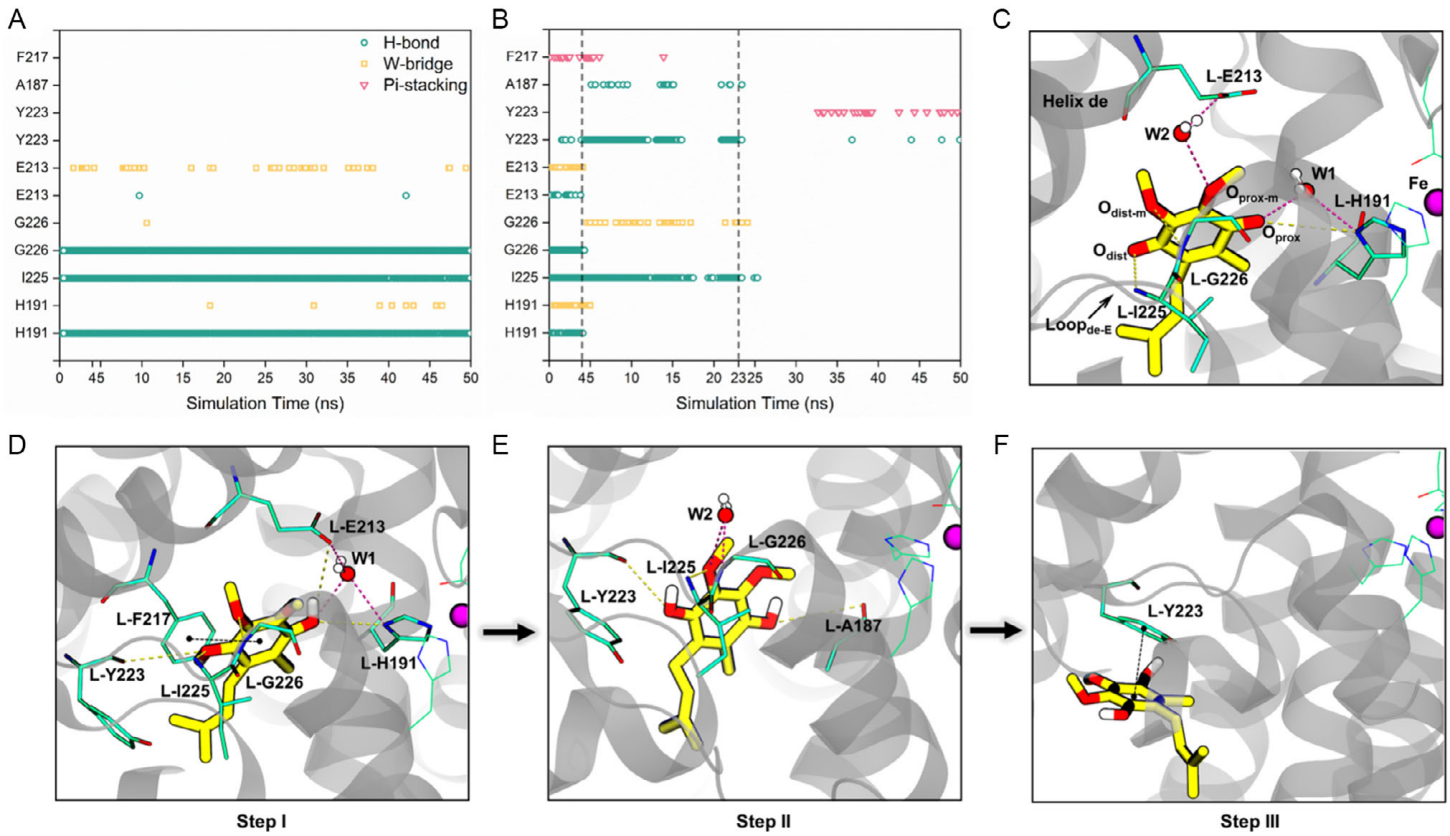
Interactions between the Q_B_/Q_B_H_2_ molecule and protein residues. A,B) The evolving interactions between the headgroups of Q_B_/Q_B_H_2_ and protein residues, respectively. C) The interactions between Q_B_ and protein residues. D–F) The evolving interactions between Q_B_H_2_ and protein residues. Hydrogen bonds and water bridges are respectively represented by yellow and purple dashed lines, while *π*‐stacking is indicated by black dashed lines. The Q_B_/Q_B_H_2_ molecule is shown in yellow, and the amino acid residues E213, G226, H191, I225, F217, Y223, and A187 on the L‐subunit are shown in green licorice. The iron ion is displayed in purple VDW models, and its coordinating residues are shown in green lines. The water molecules involved in forming water bridges are denoted as W1 and W2. O_prox_ represents the proximal oxygen atom, O_dist_ represents the distal oxygen atom, O_prox‐m_ represents the oxygen atom on the proximal methoxy group, and O_dist‐m_ represents the oxygen atom on the distal methoxy group.

To elucidate the molecular mechanisms underlying the changes in the mobility of Q_B_ and its surrounding protein environment upon reduction, we analyzed the evolution of their interactions. Prior to reduction, Q_B_ engaged in multiple interactions with the protein residues H191, I225, G226, and E213 of the L‐subunit, among which stable hydrogen bond interactions were formed with the first three residues (**Figure** **4**A,C). These interactions tether the head group of Q_B_, contributing to the overall stability of Q_B_ and surrounding protein residues (Figure 3A,C). However, the number of interactions between Q_B_H_2_ (reduced Q_B_) and protein residues gradually decreased during the course of MD simulations (Figure 4B). In the initial stage, Q_B_H_2_ formed simultaneous interactions with residues H191, I225, G226, E213, Y223, and F217, which are evenly distributed on both sides of the Q_B_H_2_ headgroup (Figure 4D). These features favoring quinol binding corresponded to a small displacement of the Q_B_H_2_ headgroup (Figure 3B,D). In the second stage, the interactions decreased exponentially, primarily on one side of the Q_B_H_2_ headgroup (Figure 4E). This led to more pronounced fluctuations in the quinol headgroup, triggering its initial dissociation. In the last stage, the complete disappearance of interactions between the Q_B_H_2_ headgroup and protein residues resulted in a “quick release” state. Eventually, the quinol headgroup only formed a *π*‐stacking interaction with Y223 of Loop_de‐E_ (Figure 4F). Furthermore, the decrease in the number of interactions consistently coincided with an increase in the distance between the Q_B_H_2_ headgroup and the iron atom (as indicated by the dashed line in Figures 3D and 4B), suggesting a correlation between them. Detailed information of the interactions between the Q_B_/Q_B_H_2_ headgroup and protein residues is provided in Supporting Information, Results and Discussions.

Taken together, prior to Q_B_ reduction, the stable interactions with specific protein residues contribute to the relatively low mobility of Q_B_. Simultaneously, these interactions play a crucial role in stabilizing the surrounding protein environment, collectively impeding the release of the Q_B_ headgroup from the binding site. However, following reduction, the gradual decrease in the number of interactions between Q_B_H_2_ and the surrounding protein residues, along with their uneven distribution, directly enhances the mobility of Q_B_H_2_. These changes not only increase the flexibility of Q_B_H_2_ movement but also trigger conformational shifts in its surrounding proteins, creating space for the subsequent release of Q_B_H_2_ headgroups. Given the conservation of the residues at the Q_B_‐binding site across different RC–LH1 structures, the aforementioned mechanism may also be relevant and extendable to other RC–LH1 from distinct species (Table S1, Supporting Information). Furthermore, it is noteworthy that during the release of the quinol headgroup, its tail remained stable due to hydrophobic interactions with the surrounding protein residues. This observation suggests that the release of the headgroup marks the initial step of the quinol dissociation process. Subsequently, the movement from the interior of the LH1 protein ring to the peripheral membrane is initiated.

### 3.2 Possible Channels for Quinol Dissociation

To unravel the dissociation channels of quinol molecules from the LH1 ring to the peripheral membrane, we performed S‐RaMD‐MD simulations (see Experimental Section for details). It was previously indicated that quinone molecules can enter and leave Photosystem II following either the head‐first or tail‐first mode.^[^ ^33^ ^]^ Therefore, in this study, we considered both dissociation modes simultaneously.

The S‐RaMD‐MD simulation trajectories indicate that quinols can successfully and exclusively dissociate through the small pores formed by adjacent LH1 α/β heterodimers, regardless of the dissociation mode employed (see Video S1, Supporting Information). This aligns with computational and recent experimental findings.^[^ ^19^, ^20^ ^]^ In 90 S‐RaMD‐MD simulations (with force‐applying atom positioned at the head and tail in 45 simulations each), there were 34 head‐first dissociation events and 39 tail‐first dissociation events (Table S2, Supporting Information). It is worth noting that quinol molecules remain undissociated only when the acceleration value was less than or equal to 0.35 kcal Å^−1 ^g^−1^, indicating the adequacy of the coverage for the acceleration parameter values in this study.

We observed that all channels determined by S‐RaMD‐MD simulations are situated within a specific region formed by LH1 subunits 15, 16, 1‐6, and the RC (Figure S3, Supporting Information). This spatial confinement might arise from hindrances between the RC and the surrounding LH1 subunits, impeding the access of quinol to the remaining area. Interestingly, in the resolved RC–LH1 structures, most quinones were also positioned in this region (Figure S3, Supporting Information).^[^ ^11^, ^12^, ^14^, ^15^, ^16^, ^17^, ^34^, ^35^ ^]^ This confirms the reliability of our current simulations and suggests that this specific region could potentially function as a cavity for tunneling quinone/quinol molecules, facilitating their movement within the LH1 ring.

**Figure 5:**
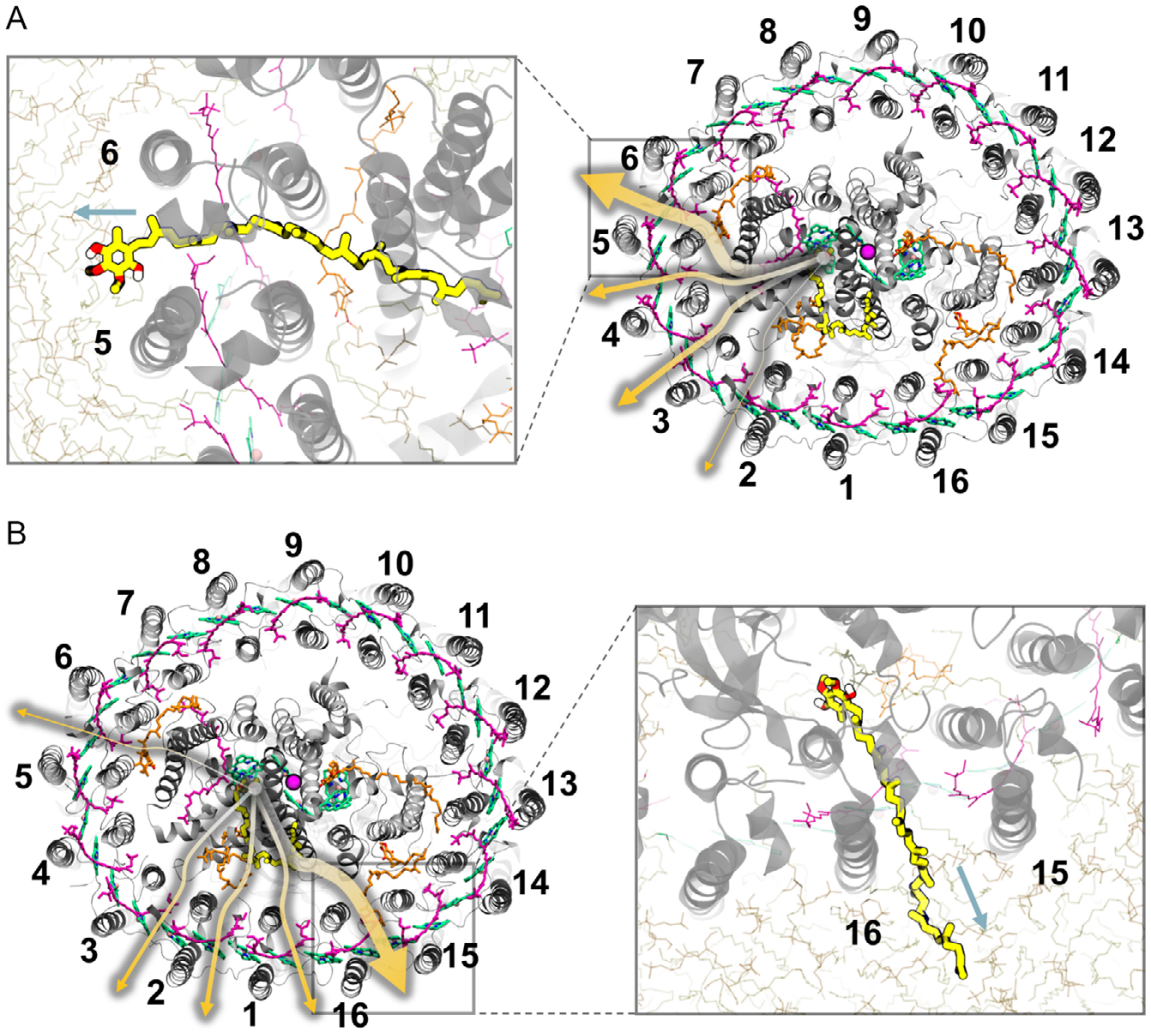
Schematic representation of the dissociation probabilities of quinol from different channels in closed RC–LH1. A) Dissociation with the head‐first mode. B) Dissociation with the tail‐first mode. The thickness of the yellow arrow represents the probability of dissociation, and the molecule presentation scheme is consistent with Figure 1.

To determine the favored dissociation channel, we calculated the percentage of quinols traversing each channel based on the associated pores. As shown in **Figure** **5**, each pore is defined by adjacent LH1 subunits on either side. For example, the most favorable pore for head‐first dissociation is between LH1 subunits 5 and 6, labeled as Channel 5‐6, which constitutes 50% of all head‐first dissociation modes. For tail‐first dissociation, the preferred one is Channel 15‐16, accounting for 46% (Figure 5, **Table** **1**).

**Table 1:** The probabilities of quinol dissociation from different channels in closed RC–LH1 ring obtained from S‐RaMD‐MD simulations. The channel with the highest dissociation probability is highlighted in bold.

Dissociation mode	Channel	Number	Possibility
Head‐first	**5‐6**	**17**	**50.0%**
	3‐4	8	23.5%
	4‐5	7	20.6%
	2‐3	1	2.9%
Tail‐first	**15‐16**	**18**	**46.2%**
	16‐1	8	20.5%
	1‐2	6	15.4%
	2‐3	5	12.8%
	5‐6	2	5.1%

While we identified preferred channels for both tail‐first and head‐first dissociations based on S‐RaMD‐MD simulations, the dissociation probabilities for these two modes are confined to internal comparisons due to their representation of distinct sampling spaces. This limited our ability to identify which channel is more favorable in terms of quinone dissociation dynamics. To address this, we employed umbrella sampling to compare the free energy profiles of the two dissociation channels.

### 3.3 Free Energy Analysis Confirms the Favorability of the Tail‐First Dissociation Channel

**Figure 6:**
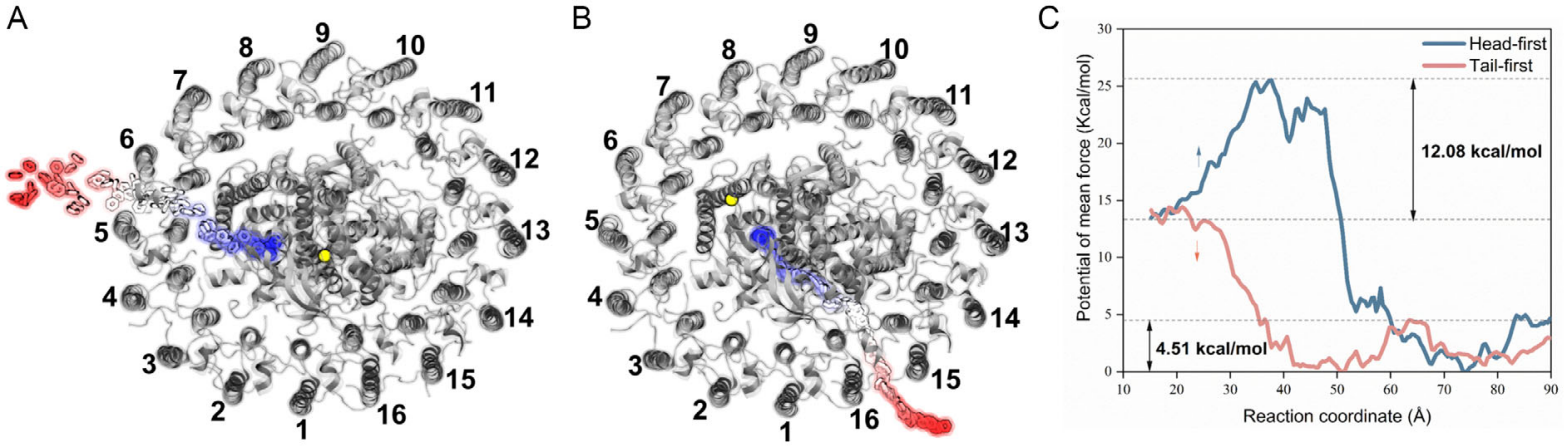
Free energy changes during the dissociation process of quinol. A,B) depict schematics of dissociation with the head‐first and tail‐first modes, respectively. As the reaction coordinate increases, the color of the six‐carbon ring of the quinol head groups gradually changes from blue to red. The yellow dots represent reference points for the reaction coordinates. C) Comparison of the free energy changes between the head‐first and tail‐first dissociation modes.

The simulation trajectories with the highest percentages for both head‐first (Channel 5‐6) and tail‐first (Channel 15‐16) dissociation were chosen to calculate the free energy profiles through umbrella sampling. The initial structure for each umbrella sampling window was selected from a snapshot obtained during the conventional MD simulation segment of the S‐RaMD‐MD simulation. Reaction coordinates were defined as the projection distance in the *x*‐*y* plane between the center of mass of the six‐carbon ring of the quinol headgroups and the Cα atoms of the chosen protein residues (L235 on the L‐subunit for the head‐first mode and W129 on the M‐subunit for the tail‐first mode) (**Figure** **6**A,B). It is noteworthy that the projected distance, rather than the direct distance, was employed as the reaction coordinate. This allows for the effective consideration of the potential movements of the Q_B_H_2_ headgroup along the membrane plane, resulting in a more accurate representation of the Q_B_H_2_ dissociation process.

First, the convergence of the PMF obtained through umbrella sampling was confirmed (Figure S4A,B, Supporting Information), demonstrating satisfactory convergence within 6 ns. Additionally, for a more intuitive comparison of the free energies of the two dissociation modes, it is essential to correct for the free energy variations between their initial and final states. Thus, this study employed the two‐end‐state free energy calculation (MM/GBSA) method, combined with the explicit membrane model, to determine the initial‐to‐final relative free energy difference (−13.70 kcal mol^−1^).

The PMF for quinol dissociation in both head‐first and tail‐first modes is depicted in Figure 6C. During the initial phase of dissociation—specifically, as Q_B_H_2_ moved from the RC to the inner ring of LH1—the tail‐first mode exhibited a prominently downhill profile, while the head‐first mode encountered an energy barrier of 12.08 kcal mol^−1^ (with reaction coordinates ranging from 15 to 50 Å for the tail‐first mode and 15–35 Å for the head‐first mode). Additionally, the maximum energy barrier for the tail‐first dissociation was 4.51 kcal mol^−1^, which is notably smaller than the corresponding barrier for the head‐first channel (12.08 kcal mol^−1^). Consequently, it is presumed that the Q_B_H_2_ molecules preferentially dissociate in the tail‐first mode, with Channel 15‐16 identified as the favorable dissociation pathway.

Consistently, the Q1 molecule was resolved near the pore 15‐16 in the cryo‐EM structure of *Rps. palustris* RC–LH1.^[^ ^17^ ^]^ Furthermore, analogous positions in the RC–LH1 complexes of other purple bacteria, such as *Tch. Tepium,* ^[^ ^11^ ^]^ *Trv. strain* 970,^[^ ^12^ ^]^ and *Blc. Viridis,* ^[^ ^16^ ^]^ also featured resolved quinone molecules (Figure S3, Supporting Information). The consistency with experimental results confirms the reliability of the dissociation channels identified in this study, indicating that the static quinone molecule revealed by cryo‐EM may represent a transient state during quinone/quinol diffusion.

### 3.4 Dynamics of Quinol Dissociation Through Channel 15‐16

**Figure 7:**
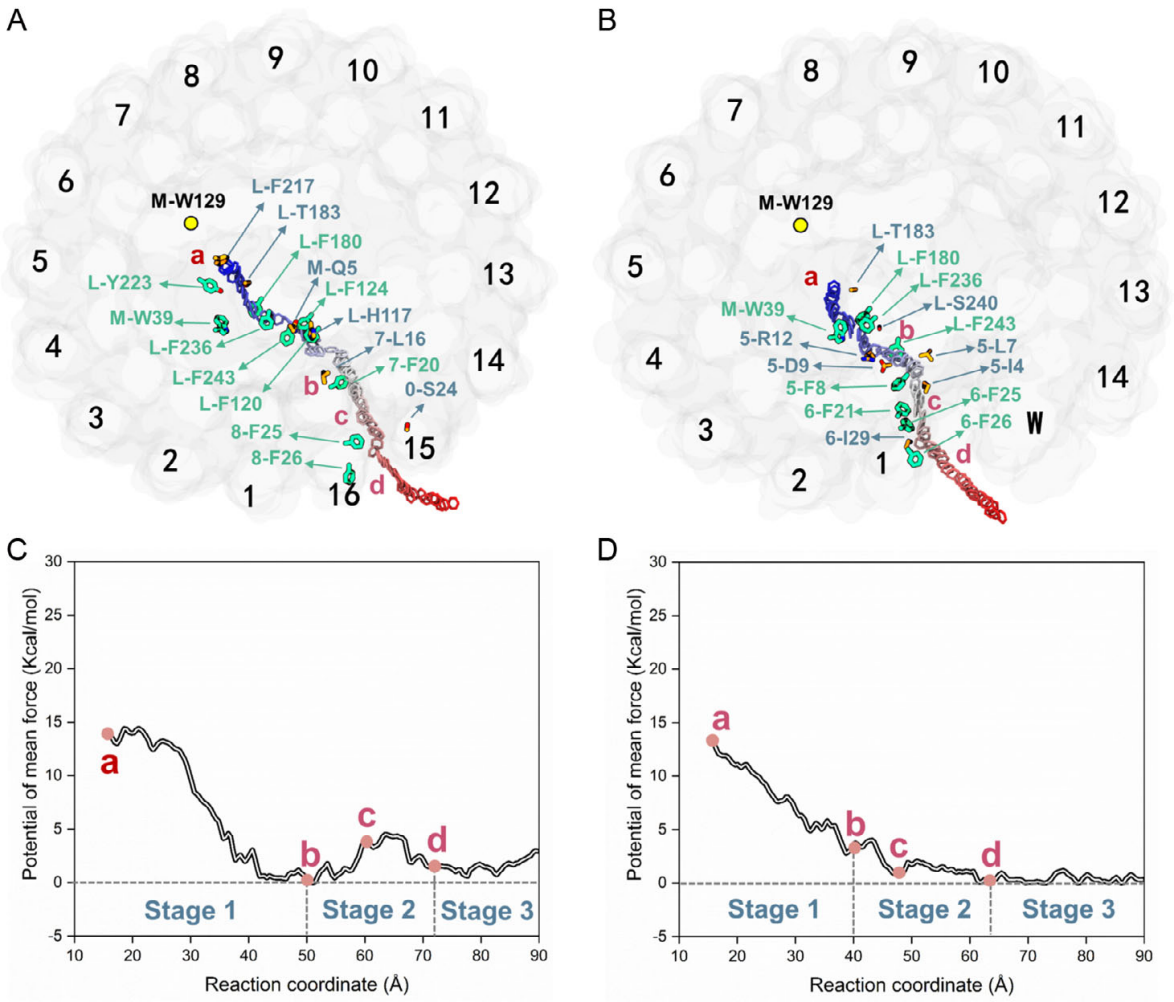
Interaction and free energy changes during the quinol dissociation process in closed and open systems. A,B) The reaction coordinates and interaction changes for quinol dissociation in closed and open RC–LH1 systems, respectively. *π*‐stacking is indicated by green font, while hydrogen bonding is marked with blue‐gray font. As the reaction coordinate increases, the color of the six‐carbon ring of the quinol head groups gradually changes from blue to red. The yellow dots represent reference points of the reaction coordinates, with both systems using M‐W129's Cα atom. C,D) The free energy changes for the closed and open RC–LH1 systems, respectively. Key reaction coordinate sites are marked with four red dots labeled as a, b, c, and d.

Based on the surrounding environment and free energy profiles of the Q_B_H_2_ headgroup, the dissociation process of the quinol molecule through Channel 15‐16 can be segmented into three stages (**Figure** **7**A,C). The first stage involves the movement of the Q_B_H_2_ headgroup inside the LH1 ring. The second stage encompasses the movement of the Q_B_H_2_ headgroup through the LH1 ring. In the third stage, the Q_B_H_2_ headgroup moves into the peripheral membrane.

During the first stage (15–50 Å), the Q_B_H_2_ headgroup initially engages in low‐occupancy *π*‐stacking with L‐Y223 (Tyr223 on the L‐subunit) (Figure 7A‐a). As the process progresses, the Q_B_H_2_ headgroup successively forms hydrogen bonds with L‐F217, L‐T183, M‐Q5, L‐H117 and 7‐L16, while interacting via *π*‐stacking with L‐Y223, M‐W39, L‐F180, L‐F236, L‐F124, L‐F243, L‐F120, and 7‐F20. These residues, particularly aromatic amino acids, are closely arranged, facilitating the movement of the Q_B_H_2_ headgroup in a “hand‐to‐hand” manner. At the end of this stage (when the reaction coordinate of umbrella sampling reaches 50 Å) (Figure 7A‐b), stable *π*‐stacking with 7‐F20 and hydrogen bonding with 7‐L16 form simultaneously, resulting in increased stability of the Q_B_H_2_ headgroup. This intensified interaction leads to a notable reduction in the free energy profiles.

The second stage (50–72 Å) can be further divided into two substages based on the interaction and free energy changes. In the first substage, the Q_B_H_2_ headgroup undergoes a process where it enters the center of the pore from the interior of LH1, ranging from 50 to 61 Å). Within the center of the pore, the Q_B_H_2_ headgroup is unable to form interactions with other amino acids (Figure 7A‐c, S5, Supporting Information), resulting in an increase in free energy profiles (4.5 kcal mol^−1^) (Figure 7C‐c,). The second substage (61–72 Å) involves the movement of the Q_B_H_2_ headgroup from the center of the pore to the peripheral membrane. The interaction between the Q_B_H_2_ headgroup and the surrounding residues shifts from low‐occupancy *π*‐stacking with 8‐F25 to stable hydrogen bonding with 0‐S24 and eventually forms high‐occupancy *π*‐stacking with 8‐F26, resulting in a downward trend in the free energy profiles. Similarly, the bell‐shaped bulge observed in the free energy profiles within the pore's center has also been previously reported.^[^ ^19^ ^]^

The last stage involves the movement of the Q_B_H_2_ headgroup in the peripheral phospholipid membrane (ranging from 72 to 90 Å), where the free energy exhibits no significant changes.

Notably, *π*‐stacking and hydrogen bonding play pivotal roles in the quinol dissociation process. The uninterrupted presence of aromatic amino acids, particularly phenylalanine, along the dissociation channel ensures that *π*‐stacking is prevalent throughout the quinol dissociation process (Figure 7A). This continuous *π*‐stacking on the dissociation channel is envisioned to guide quinol molecules in a “hand‐to‐hand” manner, essentially functioning as a conveyor belt. Intriguingly, this conveyor belt mechanism appears to be effective only after the quinone molecule is reduced to quinol (i.e., when the *p*‐benzoquinone of the headgroup is reduced to hydroquinone). At this point, the molecules become aromatic and gain the ability to engage in *π*‐stacking with aromatic amino acids (see Supporting Information Results and Discussions). This implies that the conveyor belt mechanism may specifically facilitate the dissociation of quinol, whereas other channels and mechanisms may be necessary for the entry of oxidized quinone molecules. This strategic arrangement helps mitigate “traffic jams” during quinone/quinol transport.

### 3.5 The Opening of LH1 Ring Accelerates Quinol Dissociation

The presence of the protein‐W results in an opening within the LH1 ring, allowing the RC to connect directly to the peripheral lipid membrane. To investigate the impact of this opening on the Q_B_H_2_ dissociation, the PMF of Q_B_H_2_ dissociation in the open RC–LH1 structure was calculated. As clarified above, only the tail‐first mode was taken into consideration.

**Figure 8:**
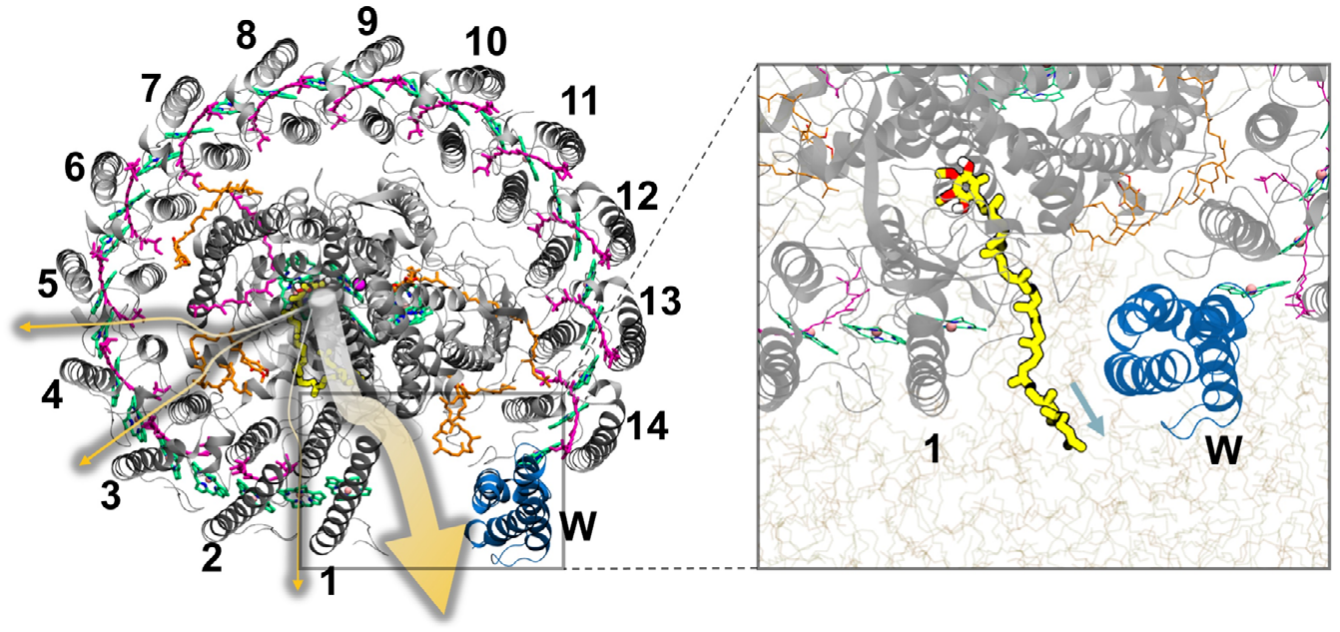
Schematic representation of the dissociation probabilities of quinol from different channels in open RC–LH1. The thickness of the yellow arrow represents the probability of dissociation, and the molecule presentation scheme is consistent with Figure 1.

The results of 45 S‐RaMD‐MD simulations on the open RC–LH1 model (Table S3, Supporting Information) revealed a notably high probability (78.9%) of Q_B_H_2_ dissociation from the opening (**Table** **2** and **Figure** **8**). This finding underscores the role of this opening as the favored channel for Q_B_H_2_ dissociation, consistent with speculations made in previous studies.^[^ ^17^ ^]^ The changes in free energy throughout the dissociation process, as illustrated in Figure 7B, were determined using the same method as in the previous section, with the corresponding validation of convergence depicted in Figure S4C, Supporting Information.

**Table 2:** The probabilities of quinol dissociation from different channels in open RC–LH1 ring obtained from S‐RaMD‐MD simulations. The channel with the highest dissociation probability is highlighted in bold.

Dissociation mode	Channel	Number	Possibility
Tail‐first	**Gap**	**30**	**78.9%**
	4‐5	3	7.9%
	1‐2	2	5.3%
	3‐4	3	7.9%

As shown in Figure 7B, the continuous evolution of hydrogen bonds and *π*‐stacking along the dissociation pathway directs the motion of the quinol headgroup though the LH1 opening, indicating that this process also follows the conveyor belt strategy (see Supporting Information, Results and Discussions).

When comparing the changes in free energy, it became evident that the second stage is mostly responsible for the difference in QBH2 dissociation between the closed and open systems (Figure S6B, Supporting Information). The free energy profiles for the former exhibit a bell‐shaped bulge with a maximum value close to 5 kcal mol^−1^, whereas those of the latter show a consistent downward trend. Structural analysis reveals significant differences in the protein structures involved in the second stage of quinol dissociation in the two systems (Figure S6A, Supporting Information). Specifically, in the closed RC–LH1 structure, a small pore is formed between adjacent LH1 heterodimers, whereas the open system contains a large gap composed of continuous phospholipid membranes formed by protein‐W. Given that the Q_B_H_2_ headgroup cannot form effective interactions with protein residues in the center of the pore (Figure S5, Supporting Information), this leads to the formation of a higher‐energy barrier in the closed system. Overall, the dissociation of Q_B_H_2_ in the open system appears to offer more advantages than that in the closed system.

The difference in the efficiency of quinol molecule dissociation based on the pores or gaps can be directly evaluated through their respective permeability coefficients. Utilizing the same methodology as previously published,^[^ ^36^ ^]^ the permeability of quinol molecules through the pores or gaps was computed. The results indicate that the permeability coefficient of quinol molecules through the small pore of the closed ring is 0.043 cm s^−1^, while that for the gap of the open ring is 0.060 cm s^−1^ (detailed information provided in Supporting Information). In comparison with the former, the rate of the latter is increased by ≈40%, signifying that Q_B_H_2_ has a higher dissociation efficiency in the open system. In other words, the opening accelerates the dissociation of Q_B_H_2_. This conclusion offers a theoretical explanation for the results, demonstrating that the opening of the ring enhances the exchange rate of ubiquinone 2 (which has a shorter isoprene tail than the natural ubiquinone 10 used in the current study) by 28 ± 5%.^[^ ^17^ ^]^ Moreover, recent transcriptomic data showed an upregulated expression of the *pufW* gene under strong illumination.^[^ ^37^ ^]^ This suggests that RC–LH1 responds to intense light, enabling some LH1 rings to open and accelerating the turnover rate of the RC.

## 4 Conclusion

By performing extensive atomic‐level theoretical simulations, we unveiled the molecular details of the quinone/quinol exchange process in the open and closed RC–LH1 structures. First, the reduction of the quinone molecule results in a significant increase in its mobility, which is closely linked to modifications in the protein structure. Second, Q_B_H_2_ exhibits a tendency to engage in dissociation in the tail‐first mode, demonstrating channel selectivity. In the context of the closed system, Q_B_H_2_ exhibits a preference for dissociation through a pore between the 15th and 16th LH1 subunits. Conversely, in the open system, Q_B_H_2_ tends to dissociate through an opening mediated by protein‐W. Finally, within the open RC–LH1 system, changes in the protein environment of the channel accelerate Q_B_H_2_ dissociation, with *π*‐stacking playing a critical role during the dissociation process. These findings enhance our theoretical understanding of the quinone/quinol trafficking mechanisms in bacterial photosynthesis. They may also inform rational design and engineering of photosynthetic systems, such as encompassing the strategic introduction of amino acid mutations, to modulate the quinone/quinol exchange rate, thereby enabling more efficient electron flux and energy conversion for bioenergy development and biotechnological applications. Moreover, the computational approaches developed in this study offer reliable toolkits for further exploration of complex biological processes.

## Conflict of Interest

The authors declare no conflict of interest.

## Author Contributions

**Jun Gao** and **Lu‐Ning Liu** contributed to idea conceptualization. **Jun Gao** and **Lu‐Ning Liu** contributed to project administration and funding acquisition. **Ruichao Mao**, **Jianping Guo**, **Lihua Bie**, and **Jun Gao** contributed in methodology and validation. **Ruichao Mao**, **Jun Gao**, and **Lu‐Ning Liu** contributed in article writing.

## Supporting information

Supplementary Material

## Data Availability

The data that support the findings of this study are available on request from the corresponding author. The data are not publicly available due to privacy or ethical restrictions.
