# Supplementary material for: Tunneling Mechanisms of Quinones in Photosynthetic Reaction Center–Light Harvesting 1 Supercomplexes
Source: Small Sci. 2024 Sep 15;4(11):2400188. doi: 10.1002/smsc.202400188 (PMC11935075; doi:10.1002/smsc.202400188)
Supplement: Supplementary file 1 — Supplementary Material [file SMSC-4-2400188-s001.zip › smsc.202400188-sup-0001-suppdata-S1.pdf]

Supporting Information

for

Tunneling Mechanisms of Quinones in Photosynthetic RC–LH1 Supercomplexes

Ruichao Mao[1,2], *Jianping Guo*[1], *Lihua Bie*[1], *Lu-Ning Liu*[1,3]*, *Jun Gao*[1]*

The file includes:

- Supplementary Figures S1 to S9
- Supplementary Tables S1 to S3
- Supplementary Methods
- Supplementary Results and Discussions
- Supplementary References

Supplementary Figures

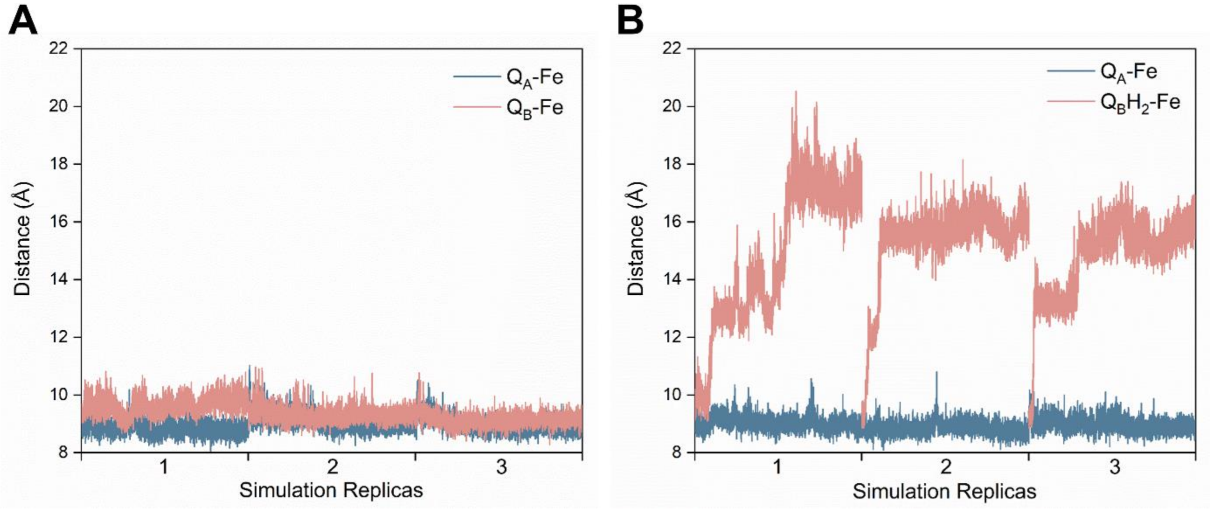

Figure S1. Distance changes between the headgroups of quinones and non-heme iron before (Q_A/Q_B) and after (Q_A/Q_BH_2) reduction. The distance change values for each state were derived from three independent replicas of MD simulations.

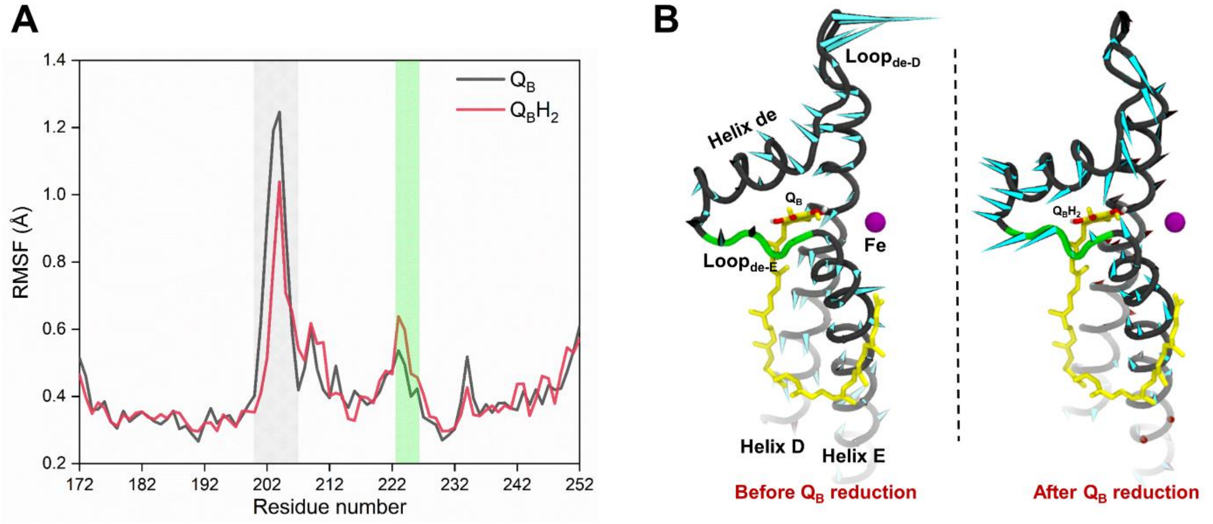

Figure S2. The mobility of protein residues around the Q_B binding site before and after Q_B reduction. (A) RMSF analysis of Cα atoms in the region spanning from Helix D to Helix E in protein chain L, including Helix D, Helix E, Helix de and two short loop regions ($Loop_{de\text{-}D}$ and $Loop_{de\text{-}E}$). The gray box corresponds to L-N200 to L-V207 ($Loop_{de\text{-}D}$), and the green box corresponds to L-Y223 to L-G226 ($Loop_{de\text{-}E}$). (B) A porcupine plot visualizing the shifts in protein residues mobility at the Q_B binding site before and after Q_B reduction. Proteins are shown in gray tube models, with the region corresponding to the green box in (A) highlighted in green. The length of the teal-colored arrows reflects the extent of movement of protein residues Cα atoms. Quinone molecules are shown in yellow, and iron ions are shown in purple.

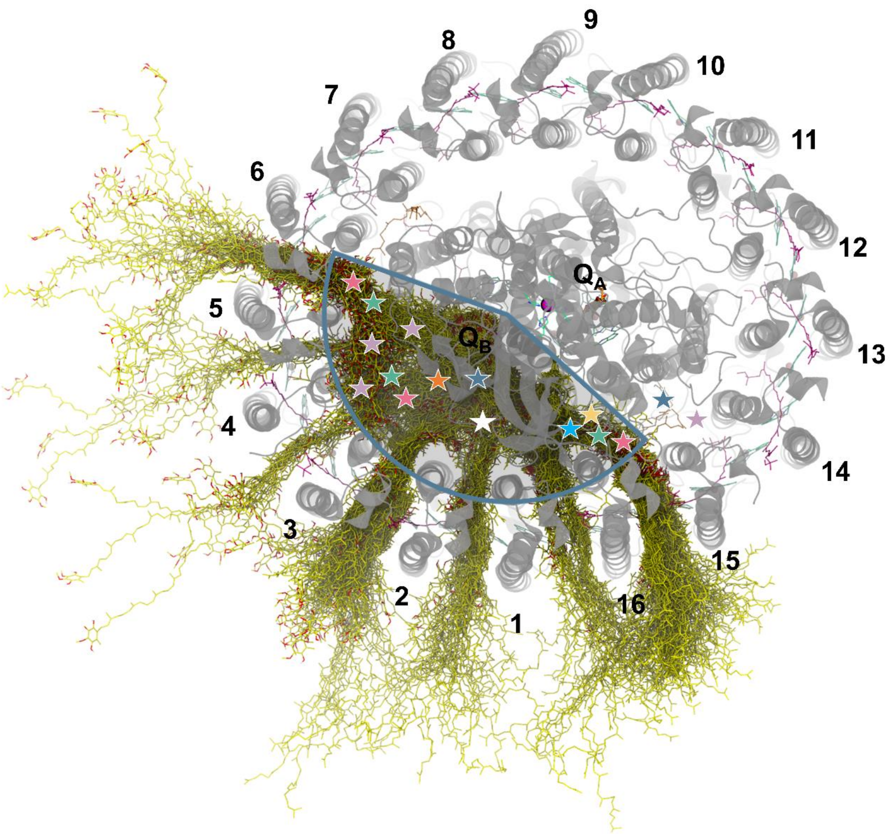

Figure S3. Potential quinone/quinol exchange cavity. Snapshots from the S-RaMD-MD simulation depicting the dissociation of quinone molecules from the reaction center are displayed in yellow licorice. The locations of experimentally resolved quinone molecules are marked with differently colored pentagrams, where *Rps. palustris* is denoted in red,[1] *Tch. tepium* in green,[2] *Trv. strain 970* in yellow,[3] *Blc. viridis* in blue,*[4]* *Rba. sphaeroides* in white,[5] *Rsp. rubrum* in orange,[6] *Rba. capsulatus* in blue-gray,[7] and *Rba. veldkampii* in purple.[8] The potential quinone/quinol exchange cavity is enclosed within a sectorial boundary.

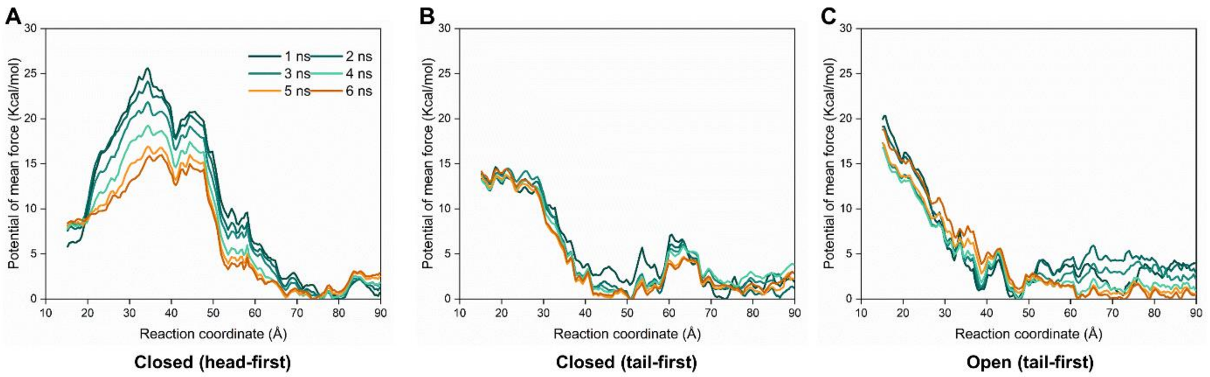

Figure S4. Convergence analysis of the free energy profiles. (A) and (B) depict the free energy changes of Q_BH_2 dissociating with the head-first and the tail-first modes in the closed RC−LH1 system, respectively. (C) represents the free energy change of Q_BH_2 dissociating with the tail-first mode in the open RC−LH1 system. The transition of the free energy profiles from blue to yellow gradually occurs as the simulation time extends.

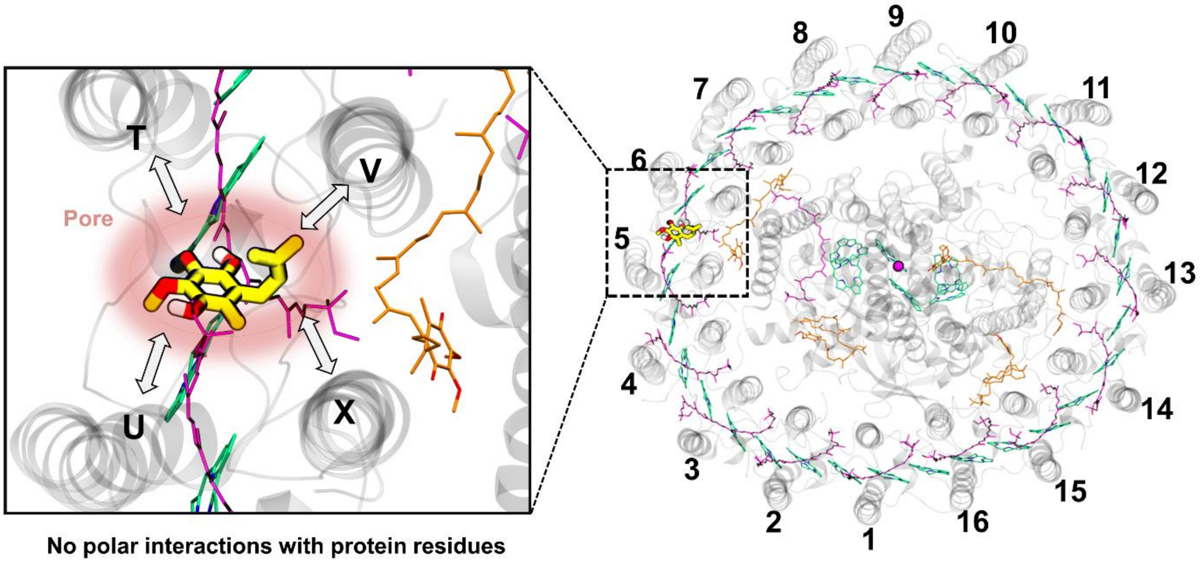

Figure S5. Schematic representation of the quinol headgroup at the center of the LH1 protein pore. The illustration demonstrates that within the pore center, the quinol headgroup is incapable of forming any polar interactions with protein residues. The center of the pore is depicted using a red ellipse. Proteins are depicted as gray transparent cartoon models, quinone molecules in orange, quinol headgroup in yellow and enclosed in an ellipse, bacterial chlorophyll in green, and carotenoids in pink.

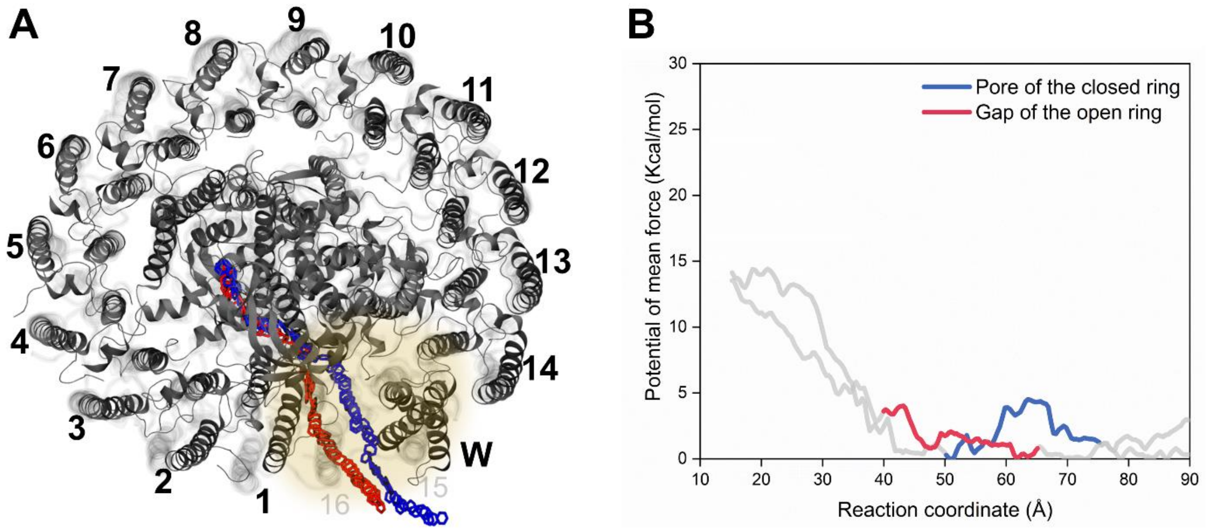

Figure S6. Comparison of quinol dissociation pathways and corresponding free energies between closed and open RC−LH1 systems. (A) Comparison of dissociation pathways between closed and open RC−LH1 systems. The open RC−LH1 is represented using a gray cartoon model, and the dissociation pathway of the six-carbon ring of the quinol headgroup is shown in red; the closed RC−LH1 is represented using a gray transparent cartoon model, and the dissociation pathway of the six-carbon ring of the quinol headgroup is shown in blue. The structural domains involved in the second stage of quinol dissociation are highlighted in yellow. (B) Comparing the PMF of quinol molecules passing through the pore of the closed ring (in blue) and the gap of the open ring (in red), with the free energy difference between the starting and ending points of the reaction coordinates uniformly corrected.

Figure S7 is included in Supplementary Methods.

Figures S8 and S9 are included in Supplementary Results and Discussions.

Supplementary Tables

Table S1. Amino acids interacting with Q_B in different RC−LH1 complexes. The columns containing the three conserved residues are displayed in different colors.

**Species**	**PDB ID**	**Resolution (Å)**	**Conserved Interacting Sites**			**Other Interacting Sites**
***Rps. palustris* (closed)**	**6Z5R**	**2.80**	**H191**	**I225**	**G226**	-
Blc. viridis	6ET5	2.87	H190	I224	G225	GLU212
Tch. tepidum	5Y5S	1.90	H199	I233	G234	ASN222
Alc. tepidum	7VRJ	2.81	H195	I229	G230	-
Trv. strain 970	7C9R	2.82	H191	I225	G226	-
Rsp. rubrum	7EQD	2.76	H191	-	G226	VAL303
Rpi. globiformis	7XXF	2.24	H191	I225	G226	-
Rba. veldkampii	7DDQ	2.84	-	-	-	VAL303
Rba. capsulatus	8B64	2.59	H190	V224	G225	-
Rba. sphaeroides	7VNY	2.79	H190	I224	G225	-
Rfl. castenholzi	5YQ7	4.10	H229	-	G259	-

Table S2. Possible channels of quinol dissociation in the closed RC−LH1 model obtained from S-RaMD-MD simulations.

Initial structure	Head-first			Tail-first		
	Parameters		Channel	Parameters		Channel
	Acce[a]	Dist[b]		Acce	Dist	
40ns	0.30	0.30	-	0.30	0.30	15-16
	0.30	0.40	5-6	0.30	0.40	16-1
	0.30	0.50	-	0.30	0.50	-
	0.35	0.30	5-6	0.35	0.30	15-16
	0.35	0.40	-	0.35	0.40	15-16
	0.35	0.50	5-6	0.35	0.50	2-3
	0.40	0.30	2-3	0.40	0.30	15-16
	0.40	0.40	3-4	0.40	0.40	15-16
	0.40	0.50	5-6	0.40	0.50	1-2
	0.45	0.30	3-4	0.45	0.30	15-16
	0.45	0.40	5-6	0.45	0.40	15-16
	0.45	0.50	4-5	0.45	0.50	2-3
	0.50	0.30	3-4	0.50	0.30	15-16
	0.50	0.40	5-6	0.50	0.40	15-16
	0.50	0.50	4-5	0.50	0.50	16-1
45ns	0.30	0.30	-	0.30	0.30	-
	0.30	0.40	-	0.30	0.40	-
	0.30	0.50	-	0.30	0.50	-
	0.35	0.30	5-6	0.35	0.30	15-16
	0.35	0.40	-	0.35	0.40	16-1
	0.35	0.50	-	0.35	0.50	2-3
	0.40	0.30	5-6	0.40	0.30	15-16
	0.40	0.40	5-6	0.40	0.40	15-16
	0.40	0.50	3-4	0.40	0.50	1-2
	0.45	0.30	4-5	0.45	0.30	16-1
	0.45	0.40	5-6	0.45	0.40	16-1
	0.45	0.50	5-6	0.45	0.50	1-2
	0.50	0.30	4-5	0.50	0.30	1-2
	0.50	0.40	4-5	0.50	0.40	5-6
	0.50	0.50	5-6	0.50	0.50	5-6
50ns	0.30	0.30	-	0.30	0.30	-
	0.30	0.40	5-6	0.30	0.40	15-16
	0.30	0.50	-	0.30	0.50	-
	0.35	0.30	-	0.35	0.30	15-16
	0.35	0.40	5-6	0.35	0.40	16-1
	0.35	0.50	5-6	0.35	0.50	1-2
	0.40	0.30	3-4	0.40	0.30	2-3
	0.40	0.40	3-4	0.40	0.40	2-3
	0.40	0.50	3-4	0.40	0.50	16-1
	0.45	0.30	5-6	0.45	0.30	15-16
	0.45	0.40	5-6	0.45	0.40	15-16
	0.45	0.50	4-5	0.45	0.50	1-2
	0.50	0.30	3-4	0.50	0.30	15-16
	0.50	0.40	5-6	0.50	0.40	16-1
	0.50	0.50	4-5	0.50	0.50	15-16

[a]accelerated speed, unit in kcal/Å/g

[b]Distance, unit in Å

Table S3. Possible channels of quinol dissociation in the open RC−LH1 model obtained from S-RaMD-MD simulations.

Initial structure	Tail-first		
	Parameters		Channel
	Acce[a]	Dist[b]	
40ns	0.30	0.30	Gap
	0.30	0.40	-
	0.30	0.50	-
	0.35	0.30	Gap
	0.35	0.40	Gap
	0.35	0.50	4-5
	0.40	0.30	Gap
	0.40	0.40	Gap
	0.40	0.50	Gap
	0.45	0.30	4-5
	0.45	0.40	1-2
	0.45	0.50	Gap
	0.50	0.30	3-4
	0.50	0.40	4-5
	0.50	0.50	3-4
45ns	0.30	0.30	-
	0.30	0.40	-
	0.30	0.50	-
	0.35	0.30	Gap
	0.35	0.40	Gap
	0.35	0.50	Gap
	0.40	0.30	Gap
	0.40	0.40	Gap
	0.40	0.50	Gap
	0.45	0.30	Gap
	0.45	0.40	Gap
	0.45	0.50	Gap
	0.50	0.30	Gap
	0.50	0.40	3-4
	0.50	0.50	Gap
50ns	0.30	0.30	Gap
	0.30	0.40	-
	0.30	0.50	-
	0.35	0.30	Gap
	0.35	0.40	Gap
	0.35	0.50	Gap
	0.40	0.30	Gap
	0.40	0.40	Gap
	0.40	0.50	Gap
	0.45	0.30	Gap
	0.45	0.40	Gap
	0.45	0.50	Gap
	0.50	0.30	1-2
	0.50	0.40	Gap
	0.50	0.50	Gap

[a]accelerated speed, unit in kcal/Å/g

[b]Distance, unit in Å

Supplementary Methods

Addition of Missing Amino Acid Residues

For the α chains in both models, all incomplete α chains (chains Y and J in the closed system; chains J and A in the open system) were completed using the chain with the highest amino acid coverage, namely M1-A46. For the β chains, all incomplete β chains (chains 2, D, 4, F, I, 8, K, 0, O, S, U, X, Q, Z, B in the closed system; all β chains in the open system) were completed using the chain with the highest amino acid coverage, namely D3-Y52. Similarly, for the reaction center proteins L, M, and H, the chains with the highest amino acid coverage were used, namely A2-P277, A2-R307, and M1-S251.

The Construction of an Open RC−LH1 Model Containing Q_B

In the cryo-EM structure of the open RC−LH1,[1] the Q_B binding site is observed to lack a quinone molecule. According to speculation,[1] the Q_B binding site in the open system is in a state where quinone molecules are prevented from entering. In comparison to the closed system, there is a conformational change in helix de (a region between helices D and E) in the open system. This conformational change lead the protein residue L-F217 on helix de occupying the Q_B binding site. Therefore, directly overlaying the Q_B molecule from the closed system onto the corresponding position in the open system would result in atomic position conflicts (Figure S7A). However, there is no significant conformational change in helices D and E at both ends of helix de, as these two regions can align well in both systems (Figure S7B). Based on this, we first replaced the protein environment (helix de) around the Q_B binding site in the open system with the corresponding part from the closed system, and then overlaid the Q_B molecule from the closed system onto the corresponding position in the open system. In this way, a reasonable construction of an open system model with a quinone molecule at the Q_B binding site has been achieved (Figure S7C).

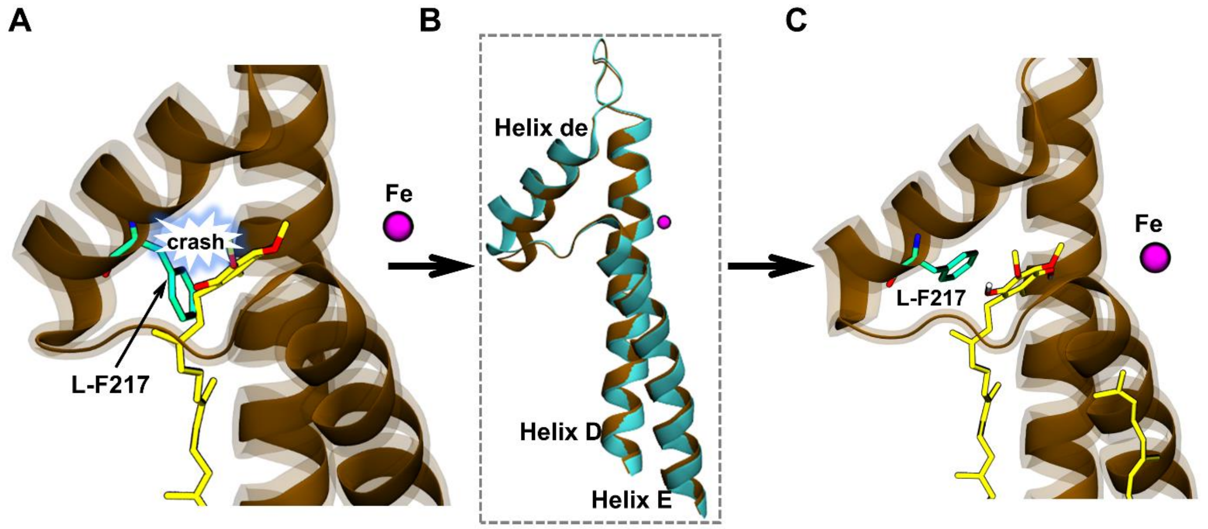

Figure S7. Construction of the open RC−LH1 model with a quinone molecule at the Q_B binding site. (A) Direct overlay of Q_B molecule from the closed system onto the corresponding position in the open system. (B) Overlay of helices D and E of chain L in the closed and open systems. The closed system is shown in teal, and the open system is shown in sienna. (C) Model of the open system with a quinone molecule at the Q_B binding site.

Force Field Parameter Setup

Standard amino acid residues were parameterized using the AMBER ff19SB force field.[9] Acidic amino acids (aspartic acid and glutamic acid) were modeled in their deprotonated form, whereas basic amino acids (lysine and arginine), excluding histidine, were protonated. For histidine residues, all were singly protonated at Nε, except those specifically coordinated with bacteriochlorophyll a (BCL) or non-heme iron (FE) through Nε, which were protonation at Nδ. For N-formylmethionine (FME), the formylated part utilized the generalized Amber force field parameter set (GAFF),[10] and the methionine part still uses the AMBER ff19SB force field parameter set. Lipid17 was employed for force field parameters of POPC, with Lipid14 being the currently published variant.[11] The parameters of BCL and bacteriopheophytin a (BPH) were taken from the study of Ceccarelli et al..[12] Previous publications[13-16] consistently stated that the non-heme iron in RC-LH1 was in the +2 oxidation state, and were magnetically coupled with two ubiquinone electron acceptors, Q_A and Q_B. Accordingly, we have considered the non-heme iron in our system as Fe^{2+}. This

approach aligns with previously simulation studies.[17, 18] In this study, to ensure the stability of Fe^{2+} and its surrounding protein environment, we modeled the non-heme iron and its five coordinating residues (M-His219, L-His231, L-His191, M-His266, and M-Glu234) as a molecular cluster. We used the method previously developed[19] to obtain the force field parameters and atomic charges for this molecular cluster, and connected the iron atom to its coordinating residues through a bonding model. For the cofactors cardiolipin (CDL), spirilloxanthin (CRT), phosphatidylglycerol (PGT), 3,4-didehydrorhodopin (QAK), quinone-10 (U10) and quinol-10 (U2H), GAFF was applied, and the atomic charges were determined by fitting the electrostatic potential around these molecules using the RESP model.[20]

Conventional RaMD and RaMD-MD Simulation Methods and Their Limitations in Describing the Quinol Dissociation Process

The random acceleration molecular dynamics (RaMD) simulation[21, 22] is a widely employed enhanced sampling method designed to expedite ligand dissociation. This method enhances the spatial sampling of the ligand by introducing a force vector ($\vec{F}$) in a random direction at the center of mass of the ligand molecule:

$$\vec{F} = k\vec{r}$$

where k is the force constant and $\vec{r}$ is a unit vector in a random direction.

Before initiating the RaMD simulation, it is necessary to designate a reference site on the receptor (top left of Figure 2, labeled R), which remains unchanged throughout the entire simulation process. Once the RaMD simulation commences, the center of mass of the ligand molecule undergoes movement under the action of a force vector $\vec{F}$ (or an acceleration a) in a random direction. After a certain number of time steps, the distance moved by the ligand’s center of mass (top left of Figure 2, labeled C) is compared to a user-defined distance threshold (d). If the former is smaller than the latter, the force $\vec{F}$ is randomly assigned a new direction; otherwise, the direction of $\vec{F}$ remains unchanged. This process iterates until the

distance between the center of mass of the ligand (C) and the initially defined reference site R exceeds another user-defined distance threshold (D), signifying a dissociation event. Thus far, the RaMD simulation method has proven effective in numerous drug design studies.[23-26] However, classic RaMD (or Pure-RaMD) simulations, due to the continuous application of an external force on the ligand molecule, may not comprehensively sample the slow motion of ligand molecules. This limitation could potentially lead to some unrealistic motions, impacting the investigation of dissociation pathways.

The RaMD-MD simulation method effectively addresses the aforementioned challenges by intermittently incorporating classical MD simulations during the RaMD simulation, proving to be a superior approach.[27-32] However, for the dissociation of quinol from RC−LH1, three specific aspects limit the application of it. Firstly, during the transition from RC to the outer cell membrane, quinol molecules may wander randomly within the LH1 protein ring, accessing different “pores” (formed by adjacent protein subunits in LH1). However, the RaMD-MD simulation algorithm tends to gradually move the center of mass of the ligand molecule away from the user-defined protein reference site R (bottom left of Figure 2), such that the dissociation of the quinol molecule is somewhat influenced by the position of R. Secondly, quinol is a long-chain molecule that passes through the pores in the LH1 protein chain in a head-first or tail-first manner.[33] The RaMD-MD simulation method applies force to the center of mass of the entire quinol molecule, making it challenging to accurately simulate the real dissociation process. Thirdly, quinol needs to maintain movement within proteins or membranes and cannot move into aqueous solution. In such cases, the conventional RaMD-MD simulation scheme becomes impractical.

Permeability calculations

Employing the same methodology as previously published,[34] the permeability of quinol molecules through pore (closed ring) or gap (open ring) was estimated. In brief, we initially calculated the position-dependent diffusion for each window of umbrella sampling (Figure S8C, S8D). Subsequently, by combining the free energy profiles (Figure S8A, S8B) with the position-dependent diffusion, local resistance was obtained (Figure S8E, S8F). Finally,

integrating the resistance for each window provided estimates for the overall permeability coefficients (0.043 cm/s for the pore, and 0.060 cm/s for the gap).

Firstly, we computed the position-dependent diffusion coefficients from molecular dynamics (MD) simulation data. For each umbrella sampling simulation window, the variance of the quinol molecule's headgroup position and the autocovariance as a function of lag time were calculated. These calculations were repeated for multiple subsamples from each trajectory. The resulting autocorrelation curves exhibited an approximately exponential decay with increasing lag time. Subsequently, the diffusion coefficient for each subsample was calculated as:

$$D(x) = \frac{var(x)}{\tau} \qquad (1)$$

Here, $var(x)$ represents the variance of the quinol molecule's headgroup position change under reaction coordinate x, and τ is the autocorrelation characteristic time. The diffusion profile (Figure S8C, S8D) is rather noisy due to the τ values, calculated by integrating the autocorrelation function. Nevertheless, τ values have small contributions to the resistance profile, which is determined mainly by the exponential free energy contribution.

The permeation coefficient P was calculated from the resistivity R,

$$\frac{1}{P} = R = \int_{x1}^{x2} R(x)\ dx \qquad (2)$$

Where $R(x)$ is the resistivity at reaction coordinate x,

$$R(x) = \frac{e^{\beta(\Delta G(x))}}{D(x)} \qquad (3)$$

Where β is the inverse of the Boltzmann constant times the temperature, and $\Delta G(x)$ is the free energy from the PMF calculations (Figure S8A, S8B).

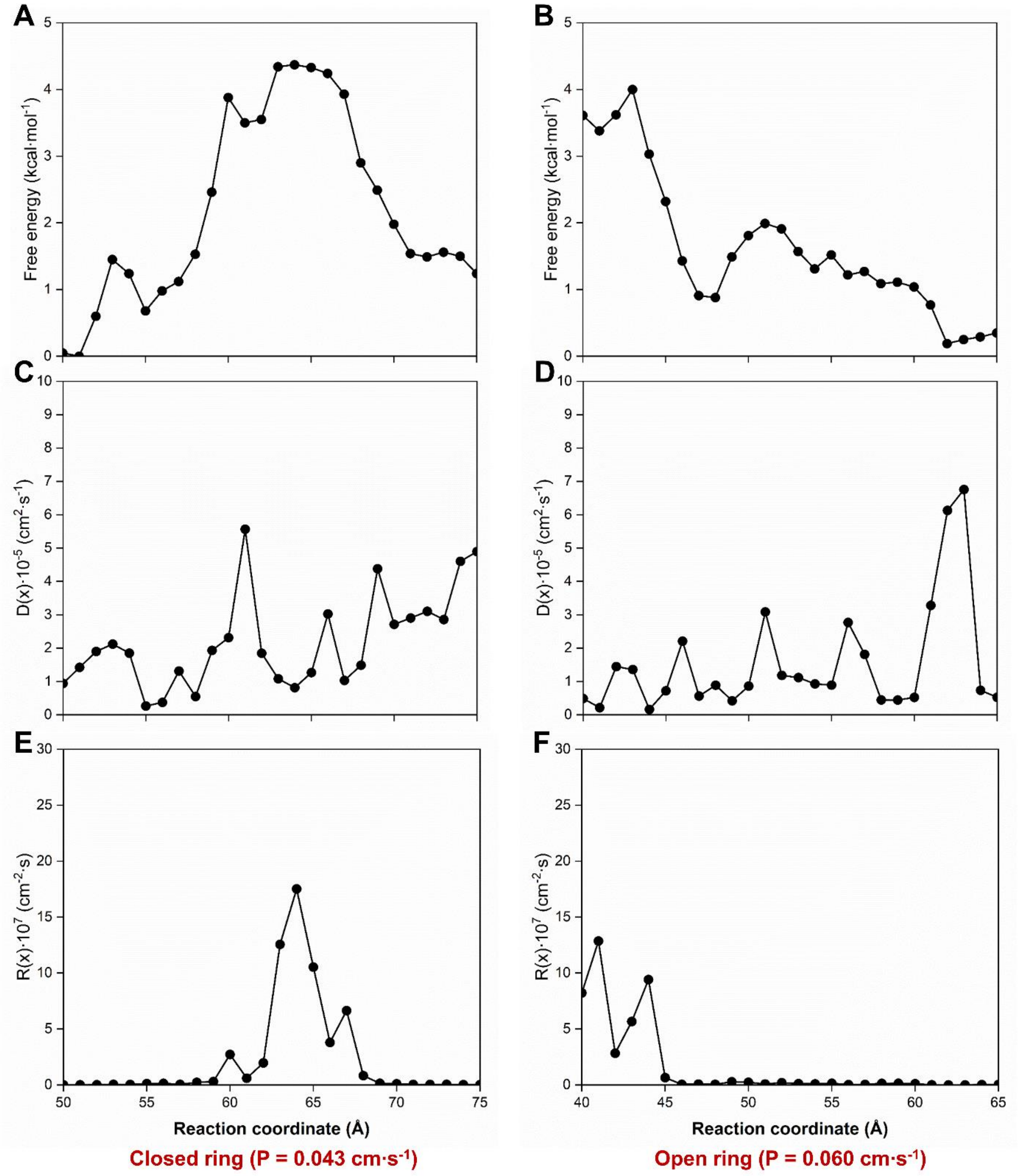

Figure S8. The permeability coefficient calculations for quinol molecules passing through the pore of the closed ring and the gap of the open ring. (A), (C) and (E) represent the free energy profiles, the local diffusion coefficients and resistance profiles corresponding to the pore of the closed ring. (B), (D) and (F) represent the free energy profiles, the local diffusion coefficients and resistance profiles corresponding to the gap of the open ring.

Supplementary Results and Discussions

Variability in the Mobility of Protein Residues at Q_B Binding Sites

The reduction of Q_B not only induces significant changes in its own position but also influences the mobility of the surrounding protein environment. Here, we conducted root mean square fluctuation (RMSF) analysis to examine the fluctuation characteristics of protein residues around the Q_B binding site. The region of interest extends from residue P172 to S252 of protein chain L, covering helix D, helix E, and helix de, as well as the two connecting loops, $loop_{de\text{-}D}$ and $loop_{de\text{-}E}$, that link the aforementioned helices (Figure S2B). In compared to the Q_B system, two segments in the Q_BH_2 system exhibit greater fluctuations. The first segment includes residues N200 to V207 (shaded region in Figure S2A), corresponding to $loop_{de\text{-}D}$. However, as $loop_{de\text{-}D}$ is distanced from the Q_B binding site, making it difficult to exert a direct impact on the quinone's motion, thus it will not be the focus of the study. The second segment comprises residues Y223 to G226 (the green boxed region in Figure S2A), corresponding to $loop_{de\text{-}E}$, coincidentally located at the Q_B binding site. Therefore, further analysis of the motion characteristics of $Loop_{de\text{-}E}$ has been carried out.

On the basis of the Cα atom motion trajectories of protein chain L before and after Q_B reduction, we employed principal component analysis (PCA) to characterize the motion features of $Loop_{de\text{-}E}$. The obtained eigenvectors represent the principal directions of protein motion, and the eigenvalues provide information about the significance of these motions along those directions. The first eigenvector obtained is the most representative, although its dominance is not particularly pronounced (explaining 16.8% of quinone motion and 25.2% of quinol motion). The hedgehog plot in Figure S2B illustrates the direction and magnitude of protein Cα motion, indicating that the motion of $Loop_{de\text{-}E}$ in the Q_B system is restricted (much smaller in magnitude compared to $Loop_{de\text{-}D}$ motion) and lacks clear directionality. However, in the Q_BH_2 system, $Loop_{de\text{-}E}$ exhibits a tendency to move away from the Q_BH_2 headgroup. Therefore, during the Q_BH_2 dissociation process, conformational adjustments occur in the protein residues of the $Loop_{de\text{-}E}$ region, creating space for the dissociation of the Q_BH_2 headgroup.

Interactions between Q_B and Surrounding Protein Residues Pre and Post-Reduction

In the closed system, the quinone molecule present in the Q_B binding site was modeled as quinone (unprotonated, Q_B) and quinol (double protonated, Q_BH_2) to simulate their oxidation and reduction states (Figure 1B).

Before reduction, the Q_B head group forms stable interactions with multiple protein residues (Figure 4A, 4C). On the proximal side, the proximal oxygen atom of the Q_B molecule's headgroup (O_{prox}) forms a stable hydrogen bond with L-H191 (H191 on the L protein chain) (Figure 4C). It is noteworthy that this interaction is strengthened by a water bridge involving a water molecule (W1) (Figure 4C). According to studies of quinone molecules in the PSII reaction center,[35, 36] it is speculated that water molecules may participate in the proton transfer processes associated with quinone molecules and histidine residues. Here, we coincidentally found a water bridge at the same position in RC−LH1, suggesting its potential role in the protonation process of the quinone molecule at the Q_B binding site. However, this conclusion requires further research for confirmation, which goes beyond the scope of the current study. Additionally, the oxygen atom of the proximal methoxy group ($O_{prox\text{-}m}$) can also form a water bridge with L-E213, mediated by a water molecule (W2) (Figure 4C). These results indicate that water bridges participate in and enhance the stability on the proximal side of the Q_B's headgroup. On the distal side, acting as the acceptor, the distal oxygen atom (O_{dist}) forms a hydrogen bond with L-I225 on $loop_{de\text{-}E}$. Similarly, the oxygen atom of the distal methoxy group ($O_{dist\text{-}m}$) serves as the acceptor, forming a hydrogen bond with L-G226 on $loop_{de\text{-}E}$. These two interactions persist throughout the course of MD simulation, stabilizing the distal side of the Q_B's headgroup (Figure 4A, 4C).

It can be observed that both on the proximal and distal sides, the interactions (especially hydrogen bond interactions) between the Q_B molecule and protein residues remain stable during the course of MD simulation. The presence of these interactions contributes to the relatively small mobility of the Q_B headgroup (Figure 3C).

After reduction, the interactions between the Q_BH_2 headgroup and protein residues

continuously evolve over the course of the simulation (Figure 4B, Figure 4D-F).

In the first stage of the Q_BH_2 headgroup exit process, the O_{prox} atom of the Q_BH_2 headgroup forms hydrogen bonds with L-H191 on helix D and L-E213 on helix de. Additionally, the interaction between the Q_BH_2 headgroup and these two residues is enhanced by a water molecule, W1, which stably resides at the center formed by the three residues. Due to the presence of W1, the Q_BH_2 headgroup can simultaneously form water bridges with L-H191 and L-E213, actively participating in the first stage of Q_BH_2 headgroup dissociation (Figure 4D). The O_{dist} atom of the Q_BH_2 headgroup primarily forms hydrogen bonds with L-I225 and L-G226, and at a lower frequency, interacts with Y223 on $loop_{de\text{-}E}$. Moreover, the six-carbon ring of the quinol head group can engage in π-stacking with the side chain of L-F217 on helix de. During this stage, multiple interactions coexist and maintain a balanced distribution. Specifically, the Q_BH_2 headgroup forms four interactions with protein residues on the proximal side, three interactions on the distal side, and one interaction involving the six-carbon ring. The temporary presence of these interactions prevents significant spatial repositioning of the headgroup at this stage (Figure 4D).

In the second stage, the hydrogen bond interaction between O_{dist} atom and L-Y223 is strengthened compared to the first stage (Figure 4B). Additionally, although the hydrogen bond between O_{dist} and L-G226 breaks, Q_BH_2 can still interact with L-G226 through a water-bridged interaction involving the $O_{dist\text{-}m}$ atom (Figure 4B, 4E). The interaction between the Q_BH_2 headgroup and L-I225 persists, while the hydrogen bond acceptor shifts from the O_{dist} atom to the adjacent $O_{dist\text{-}m}$ atom (Figure 4E). The hydrogen bond and water-bridged interactions between O_{prox} and L-His191, L-Glu213 quickly disappear, being replaced by a weak hydrogen bond interaction with A187 in Helix D (Figure 4B). Additionally, the π-stacking interaction between Q_BH_2's headgroup and L-F217 gradually diminishes during this stage. Overall, during this stage, the number of protein residues interacting with the Q_BH_2 headgroup decreases by half (4 residues) compared to the first stage (8 residues). In terms of interaction distribution, the oxygen atom on the distal side forms interactions with three protein residues (I225, G226, and Y223), while the oxygen atom on the proximal side

interacts with only one protein residue, forming a highly unstable hydrogen bond. The reduced number of interactions and their imbalanced distribution contribute to more pronounced fluctuations in the quinol headgroup compared to the previous stage (Figure 3D).

For the final stage, within the first 2 ns (23-25 ns), all interactions between the Q_BH_2 headgroup and protein residues disappear (Figure 4B), signaling that the motion of the Q_BH_2 headgroup will become more pronounced, possibly leading to further dissociation. Correspondingly, the distance between the Q_BH_2 headgroup and iron ion rapidly increases after 23 ns (Figure 3D), indicating the entry into a "quick release" state. This state persists for approximately 7 ns (23-30 ns), during which the distance between the quinol headgroup and iron ion quickly increases from 13 Å to nearly 20 Å. Subsequently, the formation of π-stacking interactions between the Q_BH_2 headgroup and L-Tyr223 (along with lower occupancy hydrogen bonding interactions) terminates this state and maintained the spatial position of the Q_BH_2 headgroup until the end of the MD simulation (Figure 4B, 4F).

Reduction of Quinone Molecules Imparts Them with Aromaticity

In this study, we employed theoretical calculations at the HF/STO-3G and B3LYP/6-31G* levels consecutively to optimize the molecular structures of quinone molecules. Subsequently, single-point energy calculations in the ground state were performed based on the optimized molecular geometries. The electrostatic potential data were then visualized by mapping them onto the isosurface of electron density, using the electron density isosurface at 0.001 a.u. as a reference for the van der Waals surface. Similar computational procedures were applied to quinol molecules.

The results from Figure S9A reveal that, due to the absence of a delocalized π bond in the six-carbon ring of the Q_B head group, the electron density at its center is relatively weak. Conversely, upon reduction to quinol (Figure S9B), the six-carbon ring in the quinol headgroup forms a delocalized π bond, leading to a significant enhancement in the electron density at the center. This implies that quinol has acquired the ability to engage in π-stacking interactions with other aromatic amino acids, such as phenylalanine, tyrosine, tryptophan, and

histidine. The pronounced increase in electron density provides a theoretical basis for the enhanced adsorption capacity of quinol in π-stacking interactions.

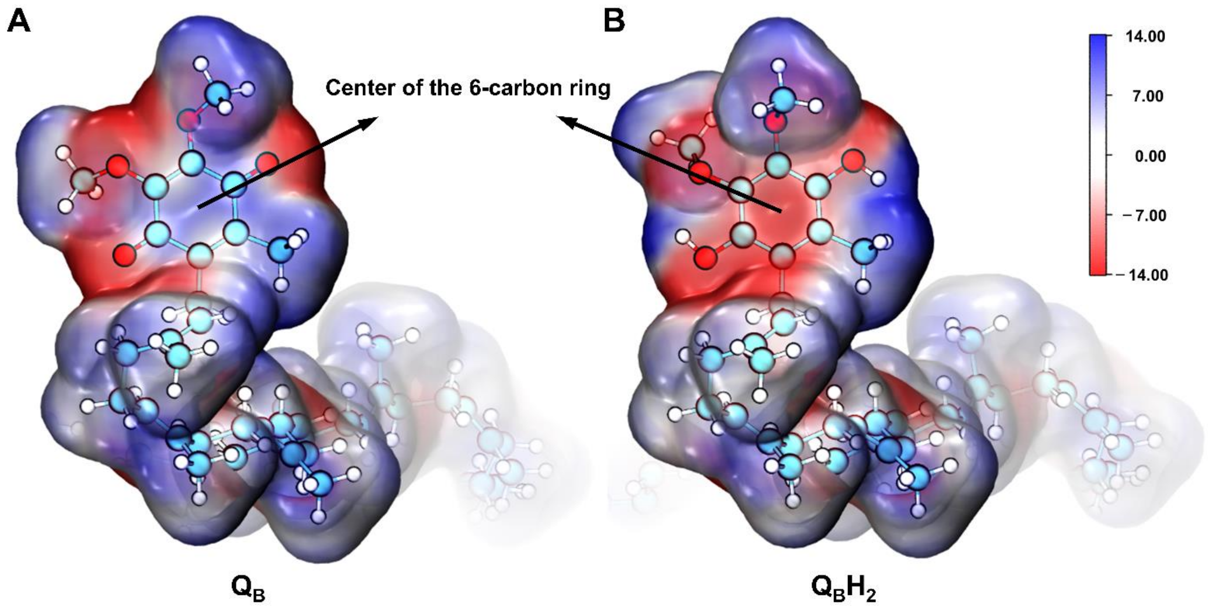

Figure S9. Comparison of molecular surface electrostatic potentials (kT/e). (A) and (B) represent the surface electrostatic potentials of quinone and quinol molecules, respectively. As the electron density increases from weak to strong, the color transitions from blue to red.

The Dynamics of Quinol Molecule Dissociation through the Gap

During the first stage (15~40 Å) of quinol dissociation in the open ring (Figure 7B, 7D), the free energy changes follow a similar decreasing trend as in the closed ring (Figure 7C, 15~50 Å). This similarity arises from the nearly identical dissociation route in both cases (Figure S6A), along which the protein environment remains similar. As the Q_BH_2 headgroup moves, it sequentially forms hydrogen bonds with L-T183, L-S240, 5-R12, 5-D9 and 5-L7, while interacting via π-stacking with M-W39, L-F180, L-F236, and L-F243. These residues guide the movement of the Q_BH_2 headgroup in a “hand-to-hand” manner. At the end of this stage (when the reaction coordinate of umbrella sampling reaches 40 Å) (Figure 7B-b), stable π-stacking (L-F243) and hydrogen bonding (5-L7) simultaneously form, enhancing the stability of the Q_BH_2 headgroup.

Similar to the closed ring, the second stage (40~63 Å) of quinol dissociation in the open ring can be divided into two sub-stages. The first sub-stage (40~46 Å) involves the process in which the Q_BH_2 headgroup passes through the gap formed by protein chain 5 and L. The

interaction between the Q_BH_2 headgroup and the surrounding environment transitions from previous hydrogen bond and π-stacking to two π-stacking interactions with 5-F8 and 6-F21. This sandwich-like stacking mode enhances the stability of the Q_BH_2 headgroup (Figure 7B-c), leading to a slight decrease in free energy profiles at this stage. In the second sub-stage (46~63 Å), the Q_BH_2 headgroup moves along protein chain 6 under the wrapping of the crowded phospholipid membrane in the gap, and there is no significant change in free energy during this stage.

In the last stage (63~90 Å), the Q_BH_2 headgroup moves in the peripheral flowing membrane, and the free energy exhibits a smooth trend.

Supplementary References

[1] D. J. K. Swainsbury, P. Qian, P. J. Jackson, K. M. Faries, D. M. Niedzwiedzki, E. C. Martin, D. A. Farmer, L. A. Malone, R. F. Thompson, N. A. Ranson, D. P. Canniffe, M. J. Dickman, D. Holten, C. Kirmaier, A. Hitchcock, C. N. Hunter, *Sci. Adv.* **2021,** *7*,eabe2631.

[2] L. J. Yu, M. Suga, Z. Y. Wang-Otomo, J. R. Shen, *Nature* **2018,** *556*,209.

[3] K. Tani, R. Kanno, Y. Makino, M. Hall, M. Takenouchi, M. Imanishi, L. J. Yu, J. Overmann, M. T. Madigan, Y. Kimura, A. Mizoguchi, B. M. Humbel, Z. Y. Wang-Otomo, *Nat. Commun.* **2020,** *11*,4955.

[4] P. Qian, C. A. Siebert, P. Wang, D. P. Canniffe, C. N. Hunter, *Nature* **2018,** *556*,203.

[5] K. Tani, K. V. P. Nagashima, R. Kanno, S. Kawamura, R. Kikuchi, M. Hall, L. J. Yu, Y. Kimura, M. T. Madigan, A. Mizoguchi, B. M. Humbel, Z. Y. Wang-Otomo, *Nat. Commun.* **2021,** *12*,6300.

[6] K. Tani, R. Kanno, X. C. Ji, M. Hall, L. J. Yu, Y. Kimura, M. T. Madigan, A. Mizoguchi, B. M. Humbel, Z. Y. Wang-Otomo, *Biochemistry* **2021,** *60*,2483.

[7] K. Tani, R. Kanno, X.-C. Ji, I. Satoh, Y. Kobayashi, M. Hall, L.-J. Yu, Y. Kimura, A. Mizoguchi, B. M. Humbel, M. T. Madigan, Z.-Y. Wang-Otomo, *Nat. Commun.* **2023,** *14*,846.

[8] L. Bracun, A. Yamagata, B. M. Christianson, T. Terada, D. P. Canniffe, M. Shirouzu, L. N. Liu, *Sci. Adv.* **2021,** *7*,1.

[9] C. Tian, K. Kasavajhala, K. a. A. Belfon, L. Raguette, H. Huang, A. N. Migues, J. Bickel, Y. Wang, J. Pincay, Q. Wu, C. Simmerling, *J. Chem. Theory Comput.* **2020,** *16*,528.

[10] J. Wang, R. M. Wolf, J. W. Caldwell, P. A. Kollman, D. A. Case, *J. Comput. Chem.* **2004,**

25,1157.
[11] C. J. Dickson, B. D. Madej, Å. A. Skjevik, R. M. Betz, K. Teigen, I. R. Gould, R. C. Walker, *J. Chem. Theory Comput.* **2014,** *10*,865.
[12] M. Ceccarelli, P. Procacci, M. Marchi, *J. Comput. Chem.* **2003,** *24*,129.
[13] L. M. Utschig, S. R. Greenfield, J. Tang, P. D. Laible, M. C. Thurnauer, *Biochemistry* **1997,** *36*,8548.
[14] E. Martin, R. I. Samoilova, K. V. Narasimhulu, T.-J. Lin, P. J. O'malley, C. A. Wraight, S. A. Dikanov, *J. Am. Chem. Soc.* **2011,** *133*,5525.
[15] M. Okamura, M. Paddock, M. Graige, G. Feher, *BBA-Bioenergetics* **2000,** *1458*,148.
[16] C. A. Wraight, *Front. Biosci.* **2004,** *9*,309.
[17] Z. Zhu, M. Gunner, *Biochemistry* **2005,** *44*,82.
[18] R. J. Wei, Y. Zhang, J. Mao, D. Kaur, U. Khaniya, M. Gunner, *Photosynth. Res.* **2022,** *152*,153.
[19] P. F. Li, K. M. Merz, *J. Chem. Inf. Model.* **2016,** *56*,599.
[20] C. I. Bayly, P. Cieplak, W. D. Cornell, P. A. Kollman, *J. Phys. Chem.* **1993,** *97*,10269.
[21] S. K. Ludemann, V. Lounnas, R. C. Wade, *J. Mol. Biol.* **2000,** *303*,797.
[22] M. Klvana, M. Pavlova, T. Koudelakova, R. Chaloupkova, P. Dvorak, Z. Prokop, A. Stsiapanava, M. Kuty, I. Kuta-Smatanova, J. Dohnalek, *J. Mol. Biol.* **2009,** *392*,1339.
[23] A.-H. Wang, Z.-C. Zhang, G.-H. Li, *Chin. J. Chem. Phys.* **2019,** *32*,277.
[24] Y. Miao, J. A. Mccammon, *Mol. Simul.* **2016,** *42*,1046.
[25] U. Doshi, D. Hamelberg, *BBA-Gen. Subjects* **2015,** *1850*,878.
[26] I. G. Rodriguez-Bussey, U. Doshi, D. Hamelberg, *Biopolymers* **2016,** *105*,35.
[27] Y. Zhao, N. Chen, C. Wang, Z. Cao, *ACS Catal.* **2016,** *6*,2145.
[28] X. Zhang, Z. Zhang, J. Guo, J. Ma, S. Xie, Y. Zhao, C. Wang, *Comput. Struct. Biotechnol. J.* **2021,** *19*,2045.
[29] Z. Zhang, F. Fan, W. Luo, Y. Zhao, C. Wang, *Front. Chem.* **2020,** *8*,730.
[30] X. Liu, Z. Zhang, N. She, J. Zhai, Y. Zhao, C. Wang, *Physical Chemistry Chemical Physics* **2022,** *24*,13806.
[31] Y. Zhao, N. She, X. Zhang, C. Wang, Y. Mo, *BBA-Proteins Proteom.* **2017,** *1865*,1020.
[32] N. She, Y. Zhao, J. Hao, S. Xie, C. Wang, *BBA-Gen. Subjects* **2019,** *1863*,609.
[33] F. J. Van Eerden, M. N. Melo, P. Frederix, X. Periole, S. J. Marrink, *Nat. Commun.* **2017,** *8*,15214.
[34] C. T. Lee, J. Comer, C. Herndon, N. Leung, A. Pavlova, R. V. Swift, C. Tung, C. N. Rowley, R. E. Amaro, C. Chipot, *J. Chem. Inf. Model.* **2016,** *56*,721.
[35] N. Kulik, M. Kuty, D. Reha, *J. Mol. Model.* **2020,** *26*,13.
[36] K. Saito, A. W. Rutherford, H. Ishikita, *Proc. Natl. Acad. Sci.* **2013,** *110*,954.
